# Bed bug preferences for host odor or aggregation odor are differentially modulated by physiological state in various odorscapes

**DOI:** 10.1002/ps.70291

**Published:** 2025-10-15

**Authors:** Ayako Wada‐Katsumata, Christopher C. Hayes, Charles A. Kwadha, Alexander Ko, Coby Schal

**Affiliations:** ^1^ Department of Entomology and Plant Pathology North Carolina State University Raleigh NC USA; ^2^ Envu Cary NC USA

**Keywords:** bed bugs, *Cimex lectularius*, aggregation, host‐seeking, odor preference, odorscape

## Abstract

**BACKGROUND:**

Bed bugs live in an ecologically and spatially restricted indoor habitat comprised of overlapping aggregation and host odors, and they traverse relatively short distances between blood‐hosts and aggregation sites. Although many studies demonstrated aggregation or host odor preference respectively, the modulation of bed bug preferences between these divergent odors is poorly understood. Given the recurrent transitions of bed bugs between replete and hungry states, we evaluated the effects of six odorscapes containing aggregation and host skin odors on bed bug preferences.

**RESULTS:**

Hunger state modulated odor preference for aggregation and foraging in all tested odorscapes. Aggregation odor attracted both fed and unfed bed bugs. Host skin odor attracted unfed bed bugs but repelled recently fed bed bugs and the addition of carbon dioxide to host odor enhanced the behavioral responses. These findings suggest that orientation to aggregation sites in fed bed bugs is driven by two distinct odor‐processing mechanisms for attractant and aversive odors. Unfed bed bugs discriminate between two attractive odors—aggregation and host odors—but host odor predominates over aggregation odor in driving their orientation behavior.

**CONCLUSION:**

Understanding the dynamic switching of odor preferences during the blood digestion cycle will guide the implementation of chemical lures in integrated pest management. Host odors alone and their co‐emission with aggregation pheromone repelled fed bed bugs from traps. Conversely, unfed bed bugs had a strong preference for host odor emitted either alone or with aggregation odor. Therefore, the independent use of either host or aggregation odor lures and their co‐emission from the same trap should be carefully considered. © 2025 The Author(s). *Pest Management Science* published by John Wiley & Sons Ltd on behalf of Society of Chemical Industry.

## Graphical abstract

The bed bug olfactory system is highly tuned to two odor sources with dedicated odor information‐processing pathways that are modulated by the satiety–hunger state. Understanding the dynamic nature of switching odor preferences at different phases of blood digestion will contribute to the development of lures with host kairomones and aggregation pheromone.

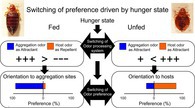

## 1 INTRODUCTION

Insects detect and differentiate semiochemicals (chemicals that influence behavior) from a dynamic background—the odorscape—composed of many other odors that vary with the local habitat, time, and the changing physiological state of the insect.^1^, ^2^ Semiochemicals within an insect's odorscape serve different ecological functions independently of their chemical nature.^3^ Examples include diverse chemicals that serve as pheromone components in intraspecific communication but also mediate cross‐species responses.^3^ Within this group are the alarm pheromones, which induce avoidance and escape behaviors in nestmates and can elicit escape in related species.^4^ Likewise, volatile sex pheromones commonly signal sexual maturity and willingness to mate but also serve in cross‐species interactions to prevent potential hybridization.^5^ On the other hand, allelochemicals convey information about the availability of a resource to the receiver such as oviposition sites and potential food sources.^3^ Because various semiochemicals (e.g., pheromones and allelochemicals) are usually admixed within an odorscape, behavioral changes in odor preferences are mediated by complex odor‐processing mechanisms including olfactory associative learning.^3^, ^6^, ^7^

The natural histories of hematogenous arthropods vary considerably among species, as do their foraging strategies. Some ectoparasites, such as lice and fleas spend most of their life on the host as they feed and reproduce.^8^, ^9^ On the other hand, in some species, such as mosquitoes, only sexually mature females blood‐feed to obtain nutrients for reproduction.^8^, ^9^ As they switch from host feeding to other behaviors (e.g., resting, oviposition, nectar feeding), the latter species are exposed to different odorscapes.^10^, ^11^, ^12^ Therefore, these species must discriminate between host kairomones, flower odors, oviposition site odors, sex pheromones and aggregation pheromones within variable odorscapes.^8^, ^9^

The common bed bug, *Cimex lectularius* (Hemiptera: Cimicidae), is unique in that all mobile stages are obligately hematophagous, wingless, and populations are ecologically and genetically fragmented within the built (indoor) environment.^13^, ^14^, ^15^ Bed bugs preferentially aggregate near, but not on, the host, and although they make excursions to the host to obtain blood meals, they return to aggregation sites daily.^13^, ^14^, ^15^ Aggregations are highly adaptive because they facilitate efficient mate finding, reproduction and foraging,^16^, ^17^ accelerate nymphal development,^18^ and offer protection from dehydration^19^ and other environmental hazards.^14^, ^15^

Early behavioral experiments on host‐seeking found that the physiological state of bed bugs affected their orientation behavior toward carbon dioxide (CO_2_), heat and host odors.^20^, ^21^, ^22^ Starved bed bugs perceive and orient toward host CO_2_ and heat at close range (a few centimeters), and bed bugs also prefer the odor of human skin, demonstrating that chemoreception is a pivotal mechanism regulating host‐seeking behavior.^20^, ^21^, ^22^ On the other hand, orientation to aggregation sites may involve negative phototaxis, thigmotaxis,^20^ and an aggregation pheromone emitted from occupied aggregation sites and the bed bug body itself.^23^, ^24^, ^25^, ^26^, ^27^, ^28^ Both skin odor components and aggregation pheromone have been characterized^27^, ^28^ and the antennal sensory responses of bed bugs to various odors and odorants also have been reported.^29^, ^30^, ^31^, ^32^, ^33^, ^34^, ^35^

Generally, in places where different types of resources are spatially separated, ecologically relevant signals in the odorscape are expected to have relatively high signal‐to‐noise ratio. However, bed bugs live in a dynamic odorscape that includes odors that are simultaneously emitted from aggregation sites and hosts,^14^, ^15^ and the distribution of these odors is frequently modified by human activity.^36^ We hypothesized that in such a composite odorscape, the switching of behavioral preferences between aggregation site attractants and host attractants might be mediated by odor discrimination mechanisms that are modulated by the physiological state of the bed bug. Although many studies have described odor preferences and antennal sensitivities in each of these two behavioral contexts independently,^27^, ^28^ it is still not known how the physiological state of bed bugs modulates their odor preferences so that adaptive aggregation and foraging behaviors are properly expressed in complex odorscapes. For example, would recently fed bed bugs accept aggregation odors that are co‐emitted and admixed with host odors? In this study, using aggregation odor that was naturally entrained on filter papers, and human skin odor swabbed onto filter papers, we conducted bioassays to evaluate the preferences of fed and starved bed bugs for these two odors, and examined how mixtures of odors that comprise the biologically relevant odorscape influence the odor preferences of bed bugs.

## 2 MATERIALS AND METHODS

### 2.1 Insects

The tested laboratory strain (Harold Harlan strain) was collected in 1973 in Fort Dix, NJ, USA and maintained on a human host until December 2008. Then, in our laboratory, it was fed on defibrinated rabbit blood until July 2021 and on human blood thereafter. It was maintained at 35–45% relative humidity (RH), 25 °C, on a 12:12 h (L:D) cycle and fed weekly on heparinized human blood (supplied by the American Red Cross under IRB #00000288 and protocol #2018‐026). We used an artificial feeding system, which has been previously described.^37^ The feeding system was housed in a North Carolina State University‐approved BSL‐2 facility (Biological Use Authorization #2020‐09‐836). Between feeding sessions, the glass feeders were sanitized with 7.5% sodium hypochlorite and 95% ethanol, and air‐dried. Because we conducted 24‐h observations in an open arena, only adult males (age unknown) were used in this study in case of accidental escape. Within 24 h post feeding, fully engorged males were separated from colony jars into groups of 100. Each group was kept in a new rearing jar (5 cm diameter × 4.5 cm height) with two clean filter paper shelters (4 × 9 cm) using #1 Whatman filter papers (Whatman, Maidstone, UK) for 2, 4, 6, 8, 10, 12 or 14 days. Tested males were discarded after a single bioassay.

### 2.2 Open arena bioassay design

**Figure 1:**
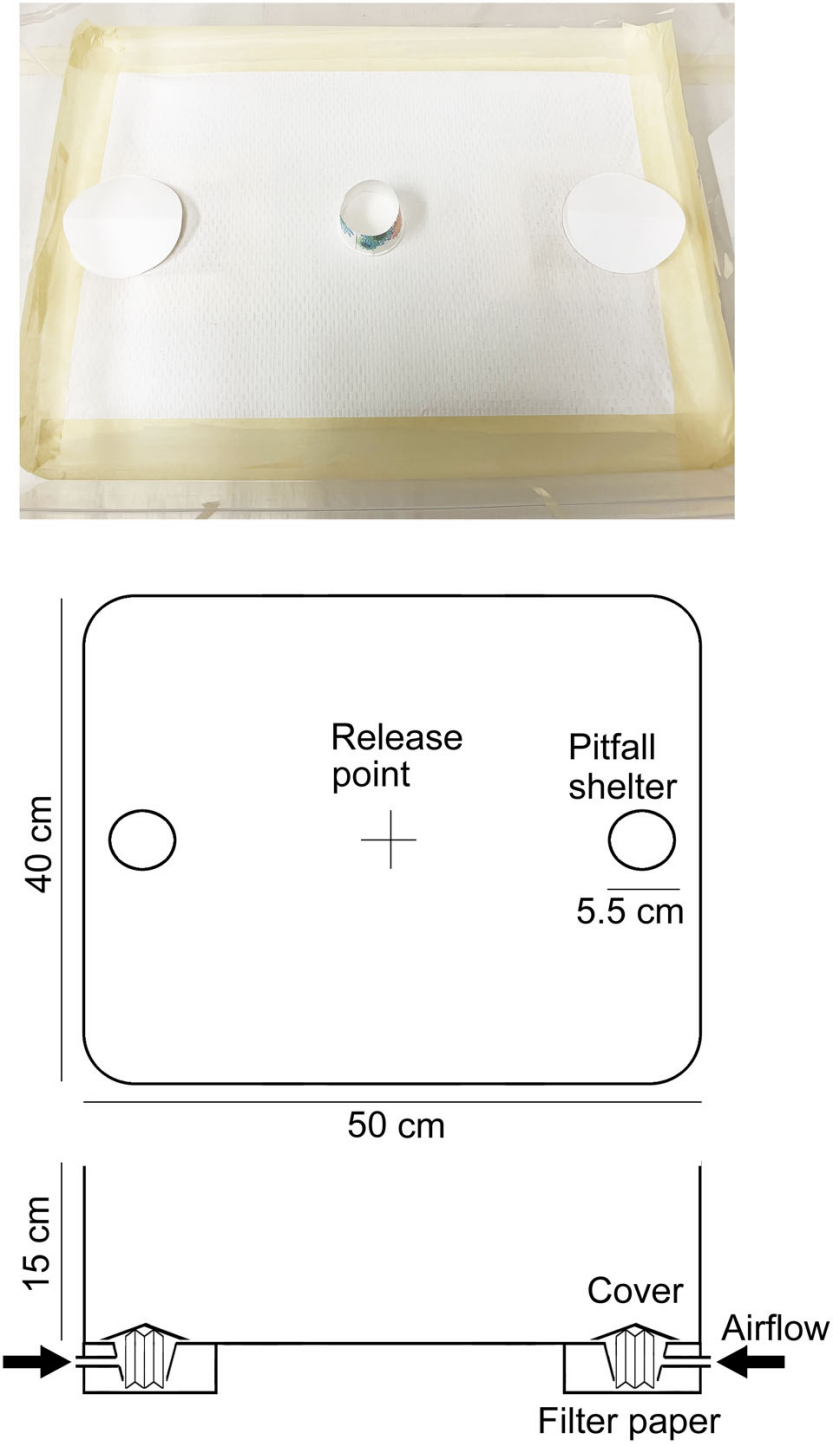
Open arena bioassay design. Bed bugs were released in the center of the arena (release point).

Arena bioassays evaluated the preferences of bed bugs for human skin odor and aggregation odor. The bottom of a plastic arena (50 cm L × 40 cm W × 15 cm H, without a lid, #2565519, Project Source, Baton Rouge, LA, USA) was lined with disposable absorbent liner (AL2050, Jaece Industries, North Tonawanda, NY, USA), secured with masking tape (2020‐48TP6, 3M Scotch, Maplewood, MN, USA), and replaced after each trial. A pitfall shelter was placed on each side of the arena, 35 cm apart (Fig. 1). Pitfall shelters were made as follows. Commercial disposable bathroom paper cups (88.7 mL, 5 cm diameter × 5.6 cm height) were placed below the arena and attached to the disposable absorbent liner so there was no gap between the cup and the liner (Fig. 1). A filter paper with a test odor (see below) was placed in each cup and the cup was lightly covered with clean filter papers during the assay to create a dark shelter within the cup. Under the dark conditions in the scotophase, the shelter did not appear to serve as a visual landmark, but it could potentially be visually detected by bed bugs during the photophase. Importantly, the shelters did not serve as traps and therefore we refer to them as pitfall shelters rather than pitfall traps; the vertical test filter paper touched the horizontal cover filter paper, so the tested bed bugs could move freely between the two shelters during each bioassay. However, we saw no evidence of bed bugs leaving a shelter after they entered it.

### 2.3 Preparation of odor sources

Bed bug‐conditioned filter papers, human skin swabs and clean filter papers were used as the aggregation odor, host skin odor and no‐odor sources, respectively. We did not carry out chemical analyses because we tested natural blends of skin and aggregation odors. For preparing the aggregation odor, two filter papers, each 4 × 9 cm made from #1 Whatman filter papers, were placed in a jar with 100 freshly fed males for 14 days. The filter papers served as substrate and shelter on which the bed bugs defecated. Because we used three arenas for each treatment, the two conditioned filter papers were cut into pieces, and an equal amount of filter paper was placed in each pitfall shelter. Thus, a single pitfall shelter contained 24 cm^2^ of bed bug‐conditioned filter paper, representing approximately 33‐male‐equivalents of aggregation odor.

For preparing the host odor, human skin was swabbed with two filter papers (#1 Whatman) following a previously validated protocol^38^ with IRB approval for recruitment, informed consent, and odor sample collections granted from North Carolina State University, Raleigh, NC, USA (IRB Approval #14173). A single adult male participant (>21 years old) provided informed consent. Prior to collecting skin swabs, he was instructed to: (i) not eat ‘spicy’ food at least 24 h before collecting a skin swab; (ii) take a morning shower; (iii) not use a deodorant or cosmetics/lotions on the sampled surfaces; (iv) not exercise or perform any strenuous physical activity; and (v) take the skin swabs 4–8 h after showering. The participant was then provided with #1 Whatman filter papers and glass vials (20 mL) and asked to collect skin swabs as follows: (i) rinse hands with water and dry before use; (ii) use a single filter paper and swab the left arm from hand to armpit for 12 s using both sides of the filter paper; (iii) rub the left leg from the lower thigh to ankle for 12 s using both sides of the filter paper; (iv) rub the left armpit for 6 s using both sides of the filter paper; (v) place the filter paper into a glass vial and label the vial; and (vi) repeat with a new filter paper swabbing the right side of the body. The papers were kept at −30 °C and tested within 24 h after collection. Three skin swab papers were cut into pieces, which were equally distributed among three pitfall shelters in three replicates. Thus, a single pitfall shelter contained the equivalent of a single filter paper swabbed on a human subject for 30 s. In Experiments 4 and 5 (see below), one pitfall shelter contained both aggregation and host odors represented by separate filter papers for aggregation odor and host odor.

### 2.4 Bioassay observations

The bioassay room was kept at 25 °C, 30–40% RH, and a photoperiod of 12 h dark (08:00 to 20:00) and 12 h light (20:00 to 08:00). These environmental conditions were the same as in the rearing environment. Bioassays were conducted for 24 h starting at 09:00. Twenty‐five males (ages unknown) were placed under an inverted cup in the center of the arena (release point, Fig. 1) and allowed to acclimate for 3 min. The cup was removed, and the positions of all bed bugs (the two pitfall shelters and arena floor) were recorded at 0, 1, 3, 6, 12 and 24 h (09:00, 10:00, 12:00, 15:00, 21:00 and 09:00). Throughout, the human observer wore a face mask and gloves to minimize bed bug exposure to human breath and skin odors, including CO_2_. Red light was used for observation during the scotophase. To avoid odor contamination among different treatments in the bioassay room, only one type of treatment (experiment) was conducted using three bioassay arenas on the same day.

### 2.5 Experimental design

We generated six types of odorscapes for six experiments, with three arenas used as three replicates in each experiment (Table 1).

**Table 1:** Experimental design of odor preferences of bed bugs in six types of odorscapes in six experiments.

	Tested males	Pitfall shelter 1^a^	Pitfall shelter 2^b^
Experiment 1	Fed (2 days post feeding)	Aggregation odor	Aggregation odor
	Unfed (14 days post feeding)	Aggregation odor	Aggregation odor
	Fed (2 days post feeding)	Host odor	Host odor
	Unfed (14 days post feeding)	Host odor	Host odor
Experiment 2	Fed (2 days post feeding)	Aggregation odor	No odor
	Unfed (14 days post feeding)	Aggregation odor	No odor
	Fed (2 days post feeding)	Host odor	No odor
	Unfed (14 days post feeding)	Host odor	No odor
Experiment 3	Fed (2 days post feeding)	Aggregation odor	Host odor
	4 days post feeding	Aggregation odor	Host odor
	6 days post feeding	Aggregation odor	Host odor
	8 days post feeding	Aggregation odor	Host odor
	10 days post feeding	Aggregation odor	Host odor
	12 days post feeding	Aggregation odor	Host odor
	Unfed (14 days post feeding)	Aggregation odor	Host odor
Experiment 4	Fed (2 days post feeding)	Host odor	Aggregation odor + Host odor
	Unfed (14 days post feeding)	Host odor	Aggregation odor + Host odor
Experiment 5	Fed (2 days post feeding)	Aggregation odor	Aggregation odor + Host odor
	Unfed (14 days post feeding)	Aggregation odor	Aggregation odor + Host odor
Experiment 6	Fed (2 days post feeding)	Aggregation odor	Aggregation odor + Host odor + CO_2_
	Unfed (14 days post feeding)	Aggregation odor	Aggregation odor + Host odor + CO_2_

^a^ Pitfall shelter 1 received either aggregation odor (bed bug conditioned filter paper) or host odor (human skin swab).

^b^ Pitfall shelter 2 received either a single odor source (aggregation or host odor) or a combination of both odors with or without CO_2_.

#### 2.5.1 Experiment 1

Both pitfall shelters had the same odor sources [either aggregation odor or host (skin) odor] to evaluate the symmetry of the bioassay environment. Two days post feeding (fed) and 14 days post feeding (unfed) adult males were used in bioassays.

#### 2.5.2 Experiment 2

Experiment 2 was a narrow odorscape in which bed bugs were given a choice between a single odor source and no odor: either aggregation or host odor *versus* clean filter paper. An odor source was placed in one pitfall shelter and clean filter paper in the other shelter. Two days post feeding (fed) and 14 days post feeding (unfed) adult males were used in bioassays.

#### 2.5.3 Experiment 3

Experiment 3 was a broader odorscape in which bed bugs were given a choice between two distinct odor sources: aggregation odor *versus* host odor. To evaluate the effect of hunger state on odor preference, separate cohorts of bed bugs were assayed 2, 4, 6, 8, 10, 12 and 14 days post feeding.

#### 2.5.4 Experiment 4

Experiment 4 was a complex odorscape in which bed bugs could choose between two admixed odor sources and a single odor source: a combination of aggregation and host odors in one pitfall shelter *versus* host odor in the other shelter. Two days post feeding (fed) and 14 days post feeding (unfed) adult males were used in bioassays.

#### 2.5.5 Experiment 5

In an inverse experiment to Experiment 4, a complex odorscape was tested in which bed bugs were given a choice between two admixed odor sources and a single odor source: a combination of aggregation and host odors in one pitfall shelter *versus* aggregation odor in the other shelter. Two days post feeding (fed) and 14 days post feeding (unfed) adult males were used in bioassays.

#### 2.5.6 Experiment 6

To add complexity to the odorscape, Experiment 5 was repeated, but with CO_2_ added to the combination of aggregation odor and host odor in one pitfall shelter *versus* aggregation odor in the other shelter. Because CO_2_ is an effective host cue in bed bugs,^39^, ^40^ we added CO_2_ to host odor to mimic more complex and realistic host cues. As shown in Fig. 1, in this experiment only, both pitfall shelters received clean air (medical quality air, Airgas Healthcare, Radnor, PA, USA) that was passed through a humidifying jar (100 mL/min for each shelter). Carbon dioxide (2% or 20 000 ppm, Airgas Healthcare) was added to the pitfall shelter that contained the aggregation and host skin odors. To avoid the accumulation of CO_2_ in the arena, observation time was limited to 0, 1, 3 and 6 h, all in the scotophase.

### 2.6 Statistical analysis

Binary choice odor preferences across multiple treatment groups were tested by full factorial repeated measures analysis of variance (ANOVA; *α* = 0.05) with Tukey's HSD in JMP (Student edition 18, Cary, NC, USA), which enabled multiple comparisons of main (fixed) effects including the number of bed bugs in each of the two pitfall shelters (Pitfall shelter main effect) across all time points (Time main effect) and the interaction of these two factors (Pitfall shelter × Time). Notably, in all except one experiment, there was a significant effect of Time (*P* ≤ 0.05). Therefore, in the main article we report the *P* values of only the main effect (Pitfall shelter). The complete test results (*F* values and df) for both main effects (Pitfall shelter, Time) and their interaction (Pitfall shelter × Time) are reported in the Data S1. When a significant interaction effect was detected, we implemented Tukey's HSD post‐hoc test, and significant differences between the two pitfall shelters by time (*P* ≤ 0.05) are indicated by an asterisk (*) on the figures and reported in the Supporting Information, Data S1.

The effects of different odorscapes on odor preferences were compared using the numbers of bed bugs recorded in pitfall shelters at the 6, 12 and 24 h time points and analyzed with one‐way ANOVA and Tukey's HSD.

## 3 RESULTS

### 3.1 Validation of bioassay conditions

**Figure 2:**
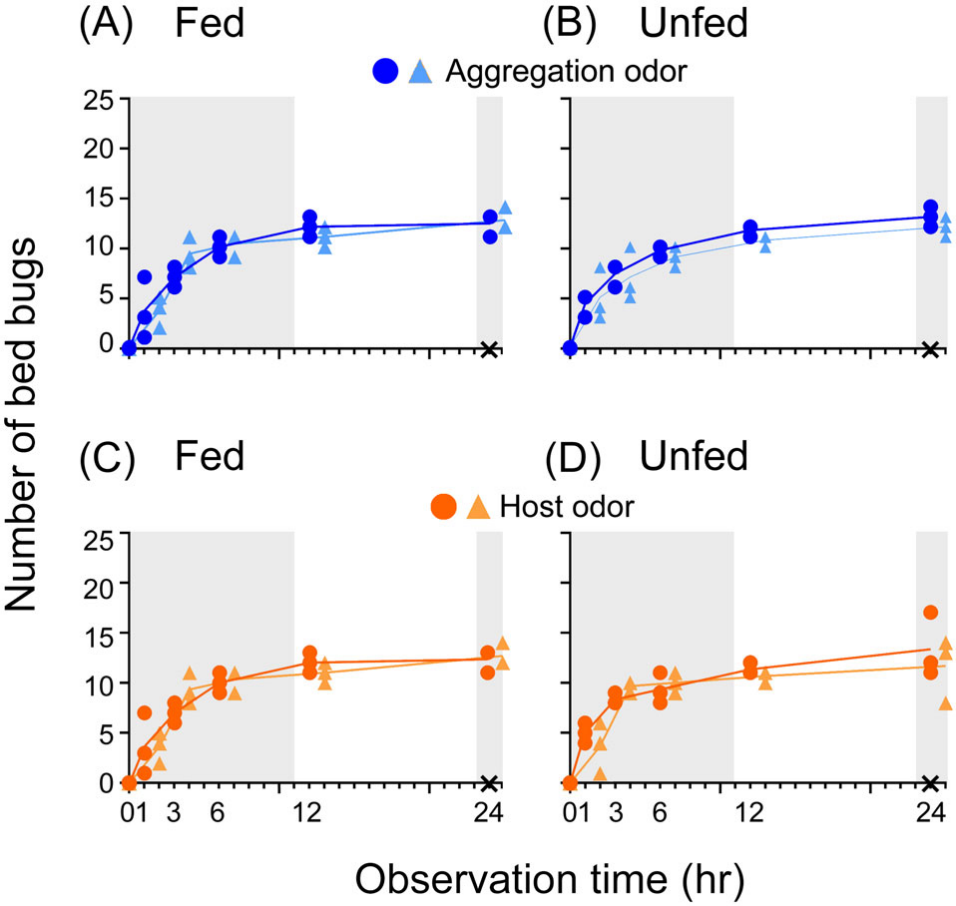
Time‐course of sheltering by fed (2 days post feeding) and unfed (14 days post feeding) bed bugs in response to aggregation and host odors. In this experiment (Experiment 1), spatial symmetry of the two stimuli within each arena was produced by placing the same odor source in both pitfall shelters. Blue (A) and light blue (B) indicate separate aggregation odor sources in fed and unfed bed bugs; orange (C) and light orange (D) indicate separate host odor sources in fed and unfed bed bugs. Each data point indicates the number of males observed in the respective pitfall shelter, showing the three replicates per treatment. The line represents the mean number of three replications. X indicates the number of non‐sheltering bed bugs at 24 h in each of the three replicates; all bed bugs were within shelters so each X represents three overlapping data points. Each replicate had 25 male bed bugs. Gray area indicates the scotophase, and the experiment started (time 0) at 09:00, 1 h after lights‐off. No bed bugs were found on the arena floor at 24 h. There was no significant difference between the two pitfall shelters in each treatment at all the observation time points (full factorial repeated measures analysis of variance, Supporting Information, Table S1).

To evaluate the symmetry of the bioassay arenas and confirm lack of position bias, bed bugs in Experiment 1 were offered either aggregation odor in both pitfall shelters or host (human) skin odor in both shelters (Fig. 2). In both treatments, both fed and unfed bed bugs sheltered equally in the two pitfall shelters with all bed bugs in all replicates sheltering within 24 h (*P* values of comparison of the two Pitfall shelters: Fig. 2(A), *P* = 0.502; Fig. 2(B), *P* = 0.638; Fig. 2(C), *P* = 0.673; Fig. 2(D), *P* = 0.742; complete test results in Supporting Information, Table S1). The results indicate that bed bugs did not discriminate between the two shelters baited with the same odor and that the bioassay arenas were symmetrical relative to cryptic (to us) environmental cues that might bias odor preferences. Notably, the results also reveal that both fed and unfed bed bugs prefer to orient toward either aggregation or host odor sources within dark shelters than to remain on the exposed arena floor.

### 3.2 Odor preferences of fed and unfed bed bugs

**Figure 3:**
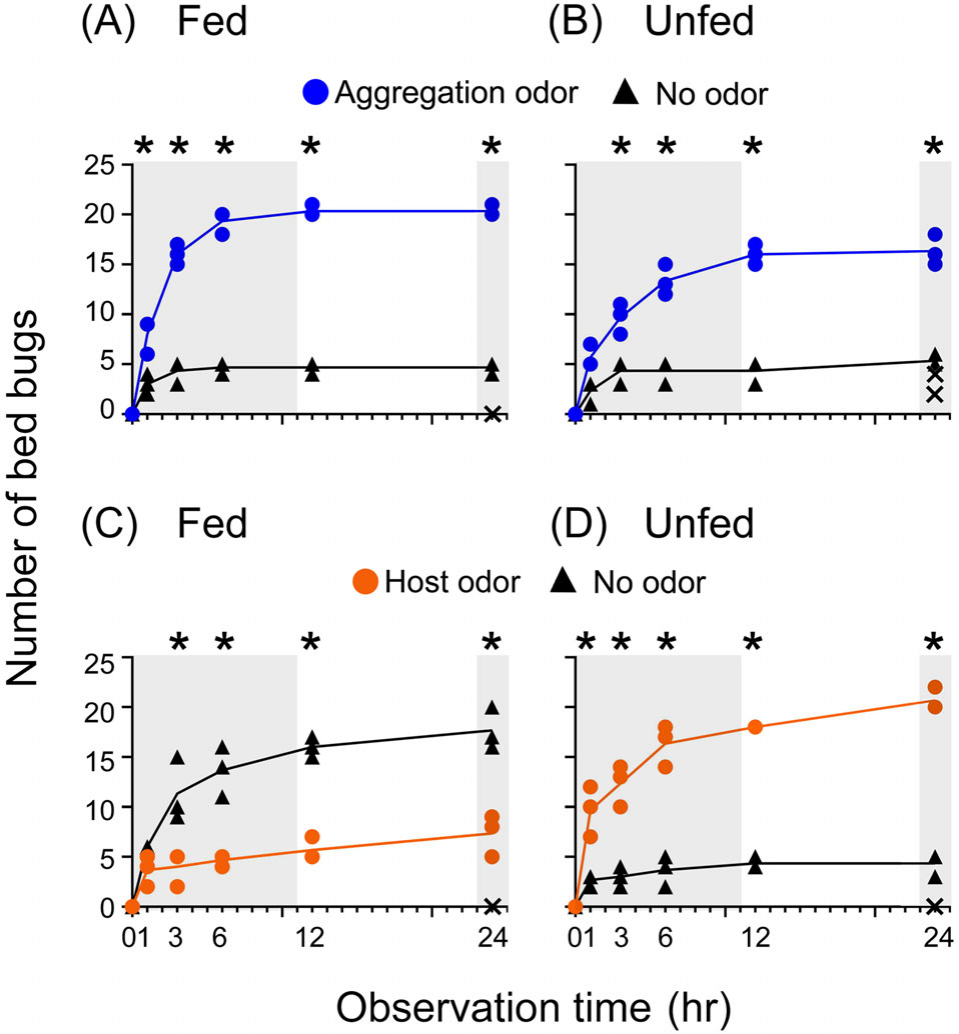
Time‐course of sheltering by fed (2 days post feeding) and unfed (14 days post feeding) bed bugs in response to aggregation and host (human) skin odors. In this experiment (Experiment 2), spatial asymmetry was introduced with an odor source in one pitfall shelter and a clean filter paper (no odor) in the other shelter. (A) Aggregation odor *versus* (B) no odor in fed and unfed bed bugs. (C) Host odor *versus* (D) no odor in fed and unfed bed bugs. Each data point indicates the number of males observed in the respective pitfall shelter, showing the three replicates per treatment. X indicates the number of non‐sheltering bed bugs at 24 h in each of the three replicates. Each replicate had 25 male bed bugs. Gray area indicates the scotophase, and the experiment started (time 0) at 09:00, 1 h after lights‐off. Asterisks at some time points indicate a significant difference between the number of bed bugs in the two pitfall shelters (full factorial repeated measures analysis of variance, Supporting Information, Table S2).

To evaluate the discrimination ability of fed and unfed males, their odor preferences were tested in an asymmetrical narrow odorscape made by pairing a single odor source (either aggregation odor or host odor) *versus* clean filter paper (no odor) (Fig. 3; Supporting Information, Table S2). Both fed and unfed bed bugs preferred the aggregation odor over no odor, but fed males made their choice faster than unfed males (*P* values comparing the two Pitfall shelters: Fig. 3(A), *P* < 0.0001; Fig. 3(B), *P* = 0.0006). However, when offered human odor *versus* no odor, the fed bed bugs preferred the clean no‐odor pitfall shelter over host odor (Fig. 3(C), *P* = 0.002). By contrast, unfed bed bugs had a strong preference for host odor over no odor (Fig. 3(D), *P* < 0.0001).

**Figure 4:**
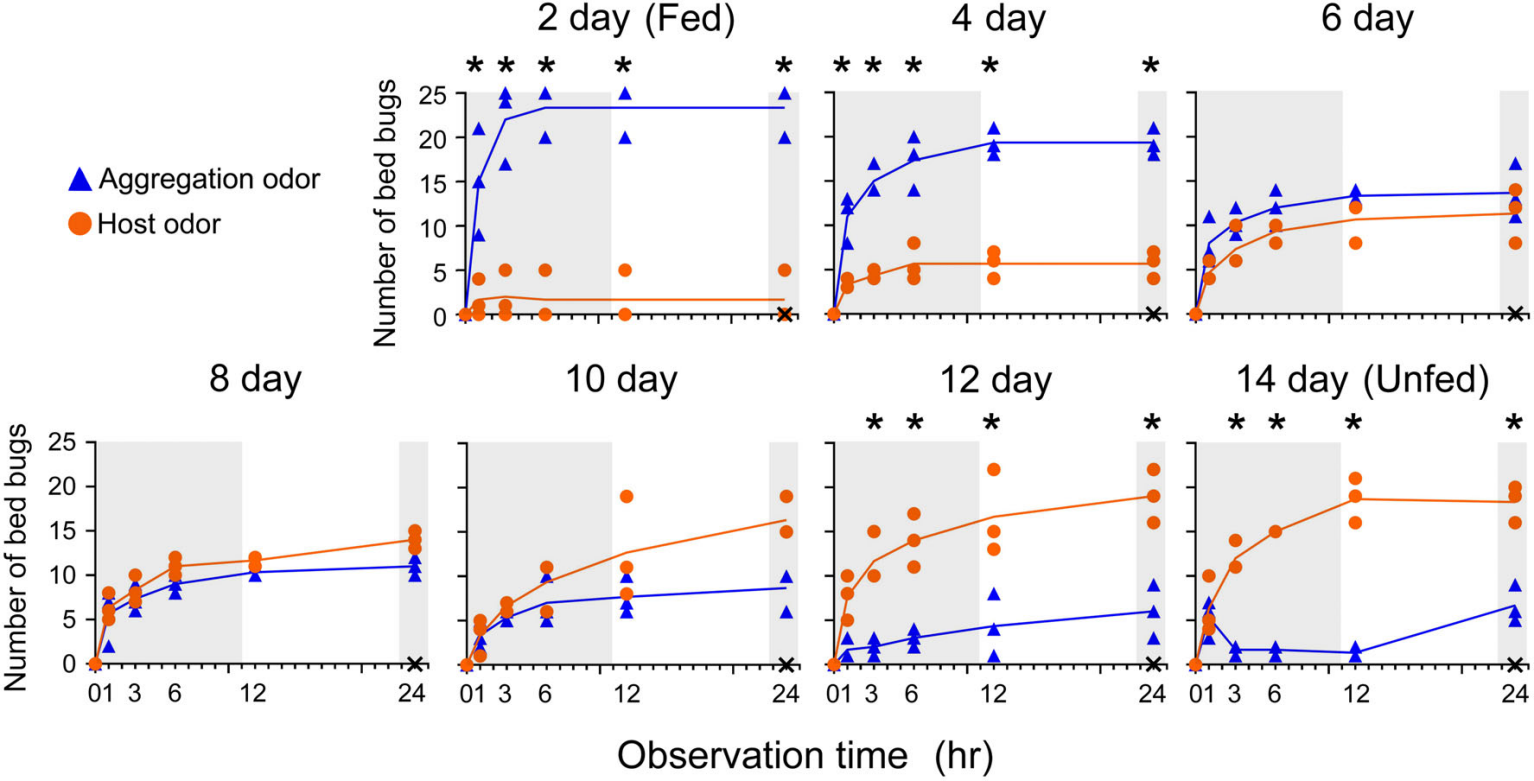
Effect of the hunger state on changes in odor preference (Experiment 3). Male bed bugs were given a choice between two pitfall shelters, one with aggregation odor and the other with host odor. Here 2, 4, 6, 8, 10 and 14 days indicate days after the blood meal. Each data point indicates the number of insects observed in the respective pitfall shelter, showing the three replicates per treatment. X indicates the number of non‐sheltering bed bugs at 24 h in each of the three replicates. Each replicate had 25 bed bugs. The gray area represents scotophase, and the experiment started (time 0) at 09:00, 1 h after lights‐off. Asterisks indicate a significant difference between the number of bed bugs in the two pitfall shelters (full factorial repeated measures analysis of variance, Supporting Information, Table S3).

To investigate the fine‐tuning of odor preferences in a broader odorscape comprised of two ecologically distinct odors, we offered bed bugs a binary choice between aggregation odor *versus* host odor, and examined their responses over time after a blood meal (Experiment 3) (Fig. 4; Supporting Information, Table S3). Satiated bed bugs, 2 and 4 days post feeding, preferred the aggregation odor over host odor (*P* values of comparisons of the two pitfall shelters: 2 days, *P* = 0.002; 4 days, *P* = 0.0006). Between 6 and 10 days post feeding, males started to shift their preferences toward host odor (6 days, *P* = 0.082; 8 days, *P* = 0.041; 10 days, *P* = 0.104). By 12 and 14 days after the blood meal, significantly more bed bugs chose the host odor over aggregation odor (12 days, *P* = 0.003; 14 days, *P* = 0.0001), with all bed bugs in all replicates sheltering within 24 h. Together with the results in Fig. 3, these results showed that recently fed bed bugs were guided by a strong preference for aggregation odor and avoidance of host odor. Unfed bed bug males that digested their previous blood meal were guided by a strong preference for human odor and lower preference for aggregation odor that increased slightly during the photophase.

### 3.3 Preferences of fed and unfed bed bugs for odor combinations

**Figure 5:**
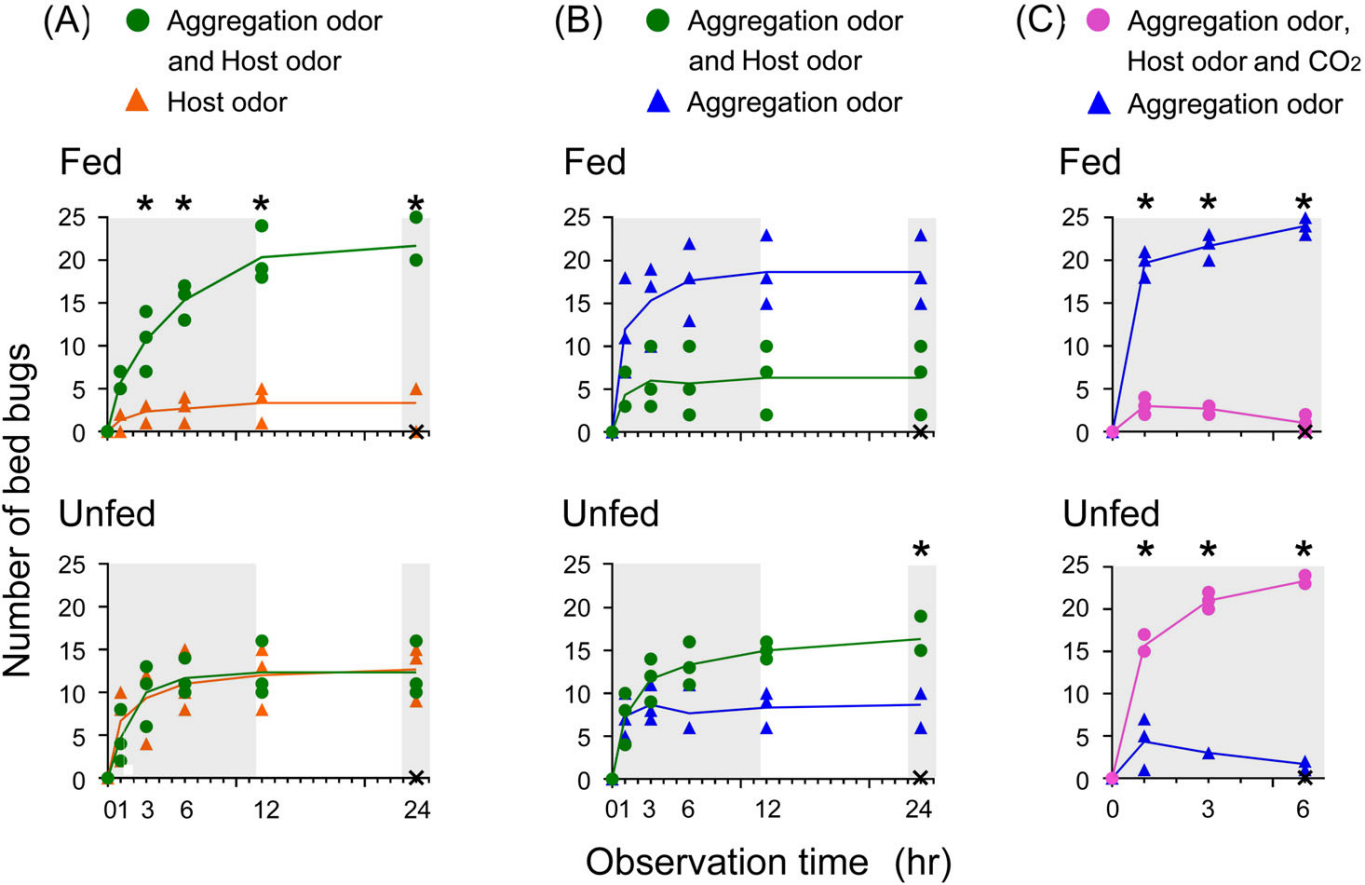
Preferences of fed (2 days post feeding) and unfed (14 days post feeding) bed bugs for odor combinations. (A) Experiment 4. A combination of aggregation odor and host odor was paired in a binary choice with host odor. (B) Experiment 5. One pitfall shelter emitted a combination of aggregation odor and host odor and the other emitted only aggregation odor. (C) Experiment 6. The same design as in Experiment 5, but CO_2_ was added to the combination of aggregation odor and host odor to enhance the quality of host‐related cues. Each data point at each observation time indicates the number of males observed in a pitfall shelter, with three replicates shown per treatment. X indicates the number of non‐sheltering bed bugs at 24 h in each of the three replicates. Each replicate included 25 bed bugs. The gray area represents scotophase, and the experiment started (time‐0) at 09:00, 1 h after lights‐off. Asterisks at some time points indicate a significant difference between the number of males in the two pitfall shelters (full factorial repeated measures analysis of variance, Supporting Information, Table S4).

To evaluate whether bed bugs discriminate aggregation odor from host odor in odor mixtures, in Experiment 4 we generated a complex odorscape with both aggregation and host odors in one pitfall shelter and host odor in the other shelter (Fig. 5(A); Supporting Information, Table S4). This situation simulates the presence of bed bug aggregations in close proximity to the host, such as on mattress seams, and the convergence of these two odor sources. All bed bugs in all replicates sheltered within 24 h. Recently fed bed bugs preferred the combination of aggregation odor and host odor over host odor alone (*P* = 0.0008). Unfed bed bugs, on the other hand, were distributed equally between the two odor sources, with no significant difference between them (*P* = 0.960). Thus, the aggregation odor did not alter the preference of hungry bed bugs for host odor (Fig. 5(A)).

In the inverse Experiment 5 we tested the preferences of fed and unfed bed bugs between a combination of aggregation and host odors in one pitfall shelter and aggregation odor in the other shelter (Fig. 5(B); Supporting Information, Table S4). Again, all bed bugs in all replicates sheltered within 24 h. Overall, fed males significantly preferred the aggregation odor alone but avoided it when it was combined with host odor (*P* = 0.031). Although there was a significant Shelter × Time interaction (*P* < 0.0001) post‐hoc tests failed to reveal differences between the two shelters at any of the time points (Supporting Information, Table S4). We recorded highly significant preferences of fed males for a shelter with aggregation odor when the other shelter contained host odor that was made more ecologically relevant with the addition of CO_2_ (*P* < 0.0001) (Experiment 6, Fig. 5C). Likewise, the preference of unfed bed bugs for host odor over aggregation odor dramatically increased in Experiment 6, when the quality of the host odor was intensified with CO_2_ (*P* < 0.0001) (Fig. 5(C)).

### 3.4 Preferences of fed and unfed bed bugs in different odorscapes

**Figure 6:**
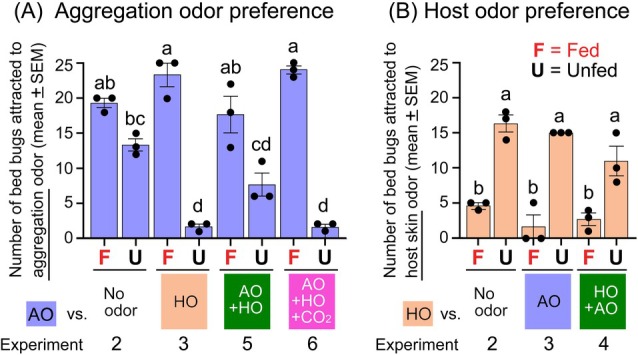
Effects of different odor combinations (odorscapes) on odor preferences of fed and unfed bed bugs at the 6 h time point. (A) Preferences for aggregation odor in Experiments 2, 3, 5 and 6. (B) Preferences for host odor in Experiments 2, 3 and 4. F, fed bed bugs 2 days post feeding; U, unfed bed bugs 14 days post feeding; AO, aggregation odor; HO, host (human skin) odor; AO + HO, combination of aggregation odor and host odor; AO + HO + CO_2_, combination of aggregation odor, host odor and CO_2_. Each data point represents the number of males observed in a pitfall shelter in one of three replicates per treatment. Different lowercase letters indicate significant differences among the treatments (one‐way analysis of variance, Supporting Information, Table S5).

Using only the 6 h time point from various experiments, we evaluated the effects of the different odorscapes on odor preferences across experiments (Fig. 6; Supporting Information, Table S5A,B). When aggregation odor was paired with various other treatments, fed and unfed bed bugs exhibited strong differential preference (one‐way ANOVA, *F*_(7,16)_ = 46.36, *P* < 0.001) (Fig. 6(A)). Thus, when aggregation odor was paired with no odor (Experiment 2), odor preference for it was slightly lower in unfed bed bugs than in fed bed bugs, with no significant difference between the two shelters. However, when the aggregation odor was paired with host odor (Experiment 3), the differential responses of bed bugs were highest because fed bed bugs were repelled by host odor and attracted to aggregation odor (push‐pull effect). Conversely, unfed bed bugs preferred host odor over aggregation odor. Fed and unfed bed bugs also discriminated between one pitfall shelter containing aggregation odor and the other emitting both aggregation and host odors (Experiment 5). However, the resolution of their behavioral responses was lower than in Experiment 3. Notably, when the pitfall shelter emitting both odors also emitted CO_2_ (Experiment 6), the high behavioral resolution of both fed and unfed bed bugs was restored as the enhanced host odor attracted unfed bed bugs and repelled fed bed bugs.

Unlike preferences for odors paired with aggregation odor, when paired with host odor the preferences of fed and unfed bed bugs were not significantly impacted by different odor combinations, or odorscapes (*P* < 0.001) (Fig. 6(B)). These results indicate that fed and unfed bed bugs are tuned to orient toward aggregation odor and host odor, respectively.

Interestingly, the same odor preference patterns were retained through the 12 and 24 h time points (Supporting Information, Table S5), suggesting that once several bed bugs entered the pitfall shelters, they might create a group effect that maintains the aggregation.

## 4 DISCUSSION

### 4.1 Hunger state modulates odor preference

Considerable progress has been made in the identification of thousands of semiochemicals, their ecologically relevant odor blends, and odor‐processing mechanisms.^3^, ^6^, ^7^ However, there is little specific understanding of how changes in the physiological state modulates odor preference for chemical cues within complex odorscapes that vary with the spatial and temporal dynamics of the local habitat.^2^ Of particular interest are situations in which different odors that drive conflicting behaviors are admixed within the odorscape, and insects must switch their responses to different odors as their physiological state changes.

The bed bug is a uniquely suitable model to investigate these sensory challenges. Intimate relationships with humans have been reported for more than four millennia^41^ and bed bugs show strong preference for human odor over other potential host odors.^22^ Bed bugs do not cover large distances for host‐seeking and in their typical odorscape host odors and aggregation odors are prominent and bed bugs must switch their preferences in relation to their satiety–hunger states while being exposed to both odors.

As in other hematophagous arthropods,^8^, ^9^ the dynamic physiological state of bed bugs rapidly changes from requiring a blood meal to being replete, and then gradually from repletion to requiring a blood meal. To understand how hunger state and odorscapes impact odor preference for adaptive orientation behaviors, we exposed recently fed and starved (unfed) bed bugs to increasingly complex odorscapes, focusing on the interaction of host odors (foraging) and aggregation odors (resting, sheltering). Our behavioral observations indicate that both fed and unfed bed bugs prefer to orient toward aggregation odor than to shelter in a clean dark shelter with no ecologically meaningful odors. However, their hunger state switches their preference for host odor, with unfed hungry males preferring host odor over aggregation odor whereas fed males appear to be repelled by it and prefer a clean shelter. This suggests that the hunger state prioritizes two odor‐processing mechanisms that mediate aggregation odor preference and avoidance response to host odor in fed bugs (≤ 4 days post feeding). In unfed bed bugs (≥ 10 days post feeding), host odor preference is prioritized by the state of hunger over anything else. These odor preferences, mediated by the satiety–hunger state, might be integrated to drive adaptive behaviors in specific odorscapes that admix host and aggregation odors. The close spatial association between bed bugs and their hosts has likely selected for their unique and narrow odor preference and behavioral responses.

There are many examples of insect behavior modulated by nutritional state, and especially by starvation.^42^, ^43^ However, the specific modulation of odor preferences and olfactory processing by the nutritional state are poorly understood. A recent study demonstrated that the nutritional state of male moths influenced their sex pheromone detection through juvenile hormone actions on the expression of odorant receptors (ORs) and odorant‐binding proteins (OBPs), highlighting a neuronal and molecular basis of a complex dietary‐dependent endocrine modulation of the peripheral olfactory system.^44^ In hematophagous insects, the blood meal causes distension of the abdomen, which suppresses host‐seeking through hormonal messengers including juvenile hormone, ecdysteroids, and neuromodulators.^45^ In kissing bugs, as in bed bugs, the time since engorgement on a blood meal profoundly affects odor preferences.^46^ Both kissing bugs and bed bugs are excellent models in which to explore such mechanisms because they undergo many recurrent cycles of hunger–satiety and modulation of their odor preferences during nymphal development and as adults.

### 4.2 Modulation of host odor preference

Hematophagous arthropods, including bed bugs, typically exploit three major host cues, processed through three sensory modalities, to identify and orient to hosts.^8^, ^9^ The first two are CO_2_ emitted during host respiration and processed through gustatory receptors (GRs),^47^ and body heat, which is detected through thermoreception; both are non‐specific cues associated with a broad range of potential hosts. Most critically, hematophagous arthropods detect, integrate, and orient toward attractive host‐specific body odors, mainly emitted from the skin; these odors are detected and processed through olfactory pathways. Host‐emitted volatiles have different profiles depending on animal species, sex, development stage, different body parts and health. More than 400 compounds have been reported on human skin.^48^, ^49^, ^50^ Between 20 and 90 volatile organic compounds (VOCs) have been detected in the headspace of the human skin surface, with most of the compounds likely of microbial or anthropogenic origin.^50^ However, not every hematophagous species detects all these odorants and shows preference for the same compounds. For example, common skin VOCs such as 1‐octen‐3‐ol, l‐lactic acid and C3–C5 carboxylic acids, are known to elicit attraction in hematophagous insects, including mosquitoes, biting midges, kissing bugs and tsetse flies.^8^, ^9^ However, these compounds are not physiologically^30^ or behaviorally^39^, ^40^ active in bed bugs, suggesting that bed bugs use a different odorant spectrum in the human odor profile compared with other hematophagous insects. Indeed, bed bugs have a low number of olfactory sensilla on their antennae,^30^, ^31^, ^32^, ^33^, ^34^, ^35^ which house fewer OBPs and ORs responsive to host VOCs than in other blood‐feeding insects such as kissing bugs (*Rhodnius prolixus*) and mosquitoes (*Anopheles gambiae*, *Aedes aegypti*).^9^ Chemical analysis and electrophysiological experiments using antennae revealed that aldehydes (C7–C10) and 6‐methyl‐5‐hepten‐2‐one (sulcatone) from skin odor were the only chemicals that stimulated olfactory receptor neurons (ORNs) of bed bugs.^31^ These odorants are detected by specific ORs (OR1, heptanal; OR36, octanal; OR1: OR36, nonanal; OR21, decanal; OR20, 6‐methyl‐5‐hepten‐2‐one).^34^ Other common skin VOCs may be detected by ionotropic receptors (IRs).^35^ However, it is still unclear how these sensitive ORNs contribute to the processing of host odors as aversive stimuli in fed bed bugs and host‐seeking in unfed bed bugs in complex odorscapes.

We also confirmed that CO_2_ enhances the quality of host cues—as in other hematophagous insects^8^, ^9^—and fine‐tunes the behavioral resolution of host odor embedded within a complex odorscape. In bed bugs CO_2_ is detected by GRs.^47^, ^51^ However, the mechanism by which CO_2_ integrates with olfactory cues and other sensory cues, which has been demonstrated in mosquitoes,^52^ remains to be explored in bed bugs. In the future it should be tested whether the modulation of host odor preference occurs at the level of differential expression of specific ORs, GRs or IRs, sensitivity of the respective receptor neurons, or integration of olfactory and gustatory information in the brain.

### 4.3 Modulation of aggregation odor preference

Bed bug aggregation is mediated by semiochemicals emitted from feces, thoracic nymphal glands, cast exuviae, and the metathoracic scent glands in adults, with all VOCs adding to the characteristic scent associated with the presence of bed bugs.^23^, ^24^, ^25^, ^26^, ^27^, ^28^ Identified aggregation pheromone components from bed bug‐conditioned substrates include dimethyl disulfide, dimethyl trisulfide, (*E*)‐2‐hexenal, (*E*)‐2‐octenal, 2‐hexanone and histamine.^23^ Whereas the first five compounds are VOCs that attract bed bugs to shelters, histamine is an arrestant that stimulates them to remain in the shelter.^23^ Electrophysiological experiments revealed ORNs and ORs for the main VOCs regulating aggregation: dimethyl disulfide and dimethyl trisulfide (OR1, OR42, OR47), (*E*)‐2‐hexenal and (*E*)‐2‐octenal (OR9b, OR11, OR15, OR17), and 2‐hexanone (OR19, OR20, OR36, OR37).^30^, ^33^, ^34^

We found that both fed and unfed bed bugs were attracted to bed bug‐conditioned filter paper containing aggregation compounds. This result supports previous studies^23^, ^24^ showing that both sexes, starved or engorged, and all stages, responded to the volatiles from bed bug‐exposed papers. It suggests that the hunger state does not switch the odor preference for aggregation odor between negative and positive responses, as it does with host odor. Instead of switching odor preference to an aversive state, the hunger state of unfed bed bugs gives lower priority for aggregation odor than host odor, resulting in higher preference for host odor. The mechanism is unknown, but in hungry bed bugs the sensory system might be less sensitive to aggregation odor or more sensitive to host odor.

Ecologically, this strategy appears to be an adaptive trait. Hungry bed bugs make short excursions to feed on the host, which requires a sensory system tuned to host odors. However, within minutes, these freshly fed bed bugs must find an aggregation site as a safe refuge where they digest the blood meal, grow, molt, mate, and oviposit. Indeed, any nocturnal excursion away from the aggregation, for whatever reason, must be swiftly followed by a return trip to the aggregation site. It logically follows that the response to aggregation odor should always be primed, whereas the response to host odor should be elevated in hungry bed bugs and completely suppressed in recently fed bed bugs.

In bed bugs, although semiochemicals and ORs involved in detection of host odors and aggregation odors have been identified, the mechanisms of odor discrimination between the blends of aggregation and host odors are unclear. There are two broad strategies of odor processing from peripheral receptors to the brain: labeled lines or combinatorial lines.^53^, ^54^ Labeled lines are composed of specific ORNs and brain regions that process behaviorally salient odors such as sex pheromones. In combinatorial lines the stimulus identity is represented in a distributed manner across a neuronal population.^53^, ^54^ It has been suggested that host‐seeking mosquitoes use combinatorial coding, because the odors of their hosts have a broad spectrum composed of a large number of compounds.^48^, ^49^, ^50^ This idea needs to be tested to understand what kind of processing strategies are used for aggregation odor and host odor in bed bugs and how satiety and hunger modulate neuronal processing.

### 4.4 Importance of understanding the odorscape for bed bug control

Bed bugs are obligately hematophagous, flightless pests of the built environment.^14^, ^15^ Unlike most hematophagous arthropods, bed bugs are not known to vector infectious diseases to their human hosts.^55^ However, their vectorial capacity should not be dismissed, as bed bugs often are associated with environments where infectious diseases flourish. In addition, their bites are associated with immune responses ranging from mild rashes to severe infectious lesions and urticaria.^56^ There is emerging evidence that histamine in their feces might be involved in allergic disease and asthma.^57^, ^58^ An important priority is innovation and development of effective surveillance and semiochemicals‐based control tools, which require better insight into the various aspects of odor‐mediated behaviors of bed bugs.

Semiochemicals that guide host‐seeking, aggregation and mating have been reported in numerous studies,^27^, ^28^ and various devices and blended odor sources are marketed to consumers and professional pest controllers.^59^, ^60^, ^61^, ^62^, ^63^ However, because of lack of understanding of behavioral switching of odor preferences and the mechanisms underlying these behavioral shifts, some devices contain blends of aggregation odor components and host odor components that may antagonize each other and may reduce trap catch. In addition, when the attractant‐based devices are placed at the wrong sites and at the wrong times, trap catch may be compromised.^64^ To meet the demand for effective lures to be used to attract host‐ and refuge‐seeking bed bugs, the blends of semiochemicals should identify, through rigorous behavioral assays, how different developmental and physiological stages respond to the synthetic blends.^59^, ^60^, ^61^, ^62^, ^63^

Our findings suggest that separate devices emitting host odors or aggregation odors should be relatively easier to engineer and deploy. Conversely, admixtures of both odors to attract host‐seeking and aggregation‐seeking bed bugs require careful attention to their specific ratios to minimize antagonistic effects, especially because host odors might adversely affect the attraction of recently fed bed bugs to aggregation odor‐based lures.

### 4.5 Limitations of this study

Our results clearly demonstrate the impacts of hunger state and odorscapes on odor preference of fed and unfed bed bugs and will guide the development of traps. Nevertheless, we acknowledge several noteworthy limitations. First, we used a laboratory‐reared strain in all our assays. It is possible that populations of *Cimex lectularius* might express variation in their odor preferences. Therefore, similar studies with freshly collected bed bugs would be informative. Second, our findings were based on open‐air arena bioassays that required considerable space and time. Therefore, we did not consider many factors that might impact the results, such as sex and development stage, concentration of odors, other cues such as heat that guide host‐seeking, and the number and composition of insects in each assay. For example, heat and CO_2_ are emitted in parallel with host odor,^20^, ^21^ so adding heat to host odor may synergize the behavioral responses, as we demonstrated with CO_2_. The assay design may also affect the results. Unlike Y‐tube olfactometers that deliver odors in directional airflow, most of our assays were conducted in still air. Therefore, whereas behaviors and choice preferences in Y‐tube olfactometers are expressed rapidly and observed in real‐time, behaviors and preferences are expressed over hours in arena assays. In addition, we used 25 bed bugs per arena assay, whereas a single subject is usually assayed in Y‐tube assays. In such protracted observation of groups of insects, interactions among individuals may influence their choices over time.^20^, ^21^ Bed bugs prefer to maintain close contact with conspecifics and surfaces within their refuges^20^, ^21^, ^22^ so social interactions might be particularly important in assays with this species. In our observation, most insects made a shelter choice within 6 h and their choice remained relatively constant for 24 h. We did not record movement patterns between pitfall shelters, but it is possible that bed bugs tended to remain in occupied pitfall shelters. These differences in assay conditions may produce variance and even discrepancy between results from Y‐tube^64^ and arena assays (this paper).

## 5 CONCLUSIONS

Switching of odor preferences is commonly observed in animals and this behavior is supported by integrated odor processing in the peripheral and central nervous systems, which in turn is influenced by environmental and physiological factors. Although understanding the modulation of odor preferences is important to support the development of semiochemicals‐based pest control tools, most studies have focused on single behaviors such as host‐seeking, mating, and aggregation, and analyzed odor processing either in the peripheral sensory system or the brain. We exposed bed bugs to odorscapes consisting of different combinations of aggregation and host odors and demonstrated that hunger state modulates bed bug host‐seeking and aggregation behaviors. Most importantly, our observations revealed that odors are encoded as ‘attractive’ or ‘aversive’ depending upon the satiety–hunger state and ecological context, highlighting that integration of sensory information and physiological state can change the valence of odors to elicit orientation to one odor and aversion to another odor. Understanding how to manipulate the processing of olfactory information may support innovative design of bed bug control devices.

## CONFLICT OF INTEREST

The authors declare no conflict of interest.

## Supporting information


**Data S1.** Supporting Information.

## Data Availability

The data that support the findings of this study are openly available in DRYAD at https://datadryad.org/, reference number DOI: 10.5061/dryad.nzs7h4529.

## References

[1] von Uexküll JJ , Umwelt und Innenwelt der Tiere, in Klassische Texte der Wissenschaft, ed. by Mildenberger F and Herrmann B . Springer‐Verlag, Berlin, Germany (2014).

[2] Conchou L , Lucas P , Meslin C , Proffit M , Staudt M and Renou M , Insect odorscapes: from plant volatiles to natural olfactory scenes. Front Physiol 10:972 (2019). 31427985 10.3389/fphys.2019.00972 PMC6688386

[3] Wyatt TD , Pheromones and animal behavior: chemical signals and signatures. Cambridge University Press, New York, USA (2014).

[4] Kannan K , Galizia CG and Nouvian M , Olfactory strategies in the defensive behaviour of insects. Insects 13:4702022 (2022). 10.3390/insects13050470 PMC9145661 35621804

[5] Yang JC , Zhang JP , Wu CY , Bai Y , Guedes RNC , Dewer Y *et al*., Diversity and role of volatile terpene and terpenoid pheromones in insects. J Econ Entomol 118:9–18 (2024). 10.1093/jee/toae271 39578941

[6] Souto PM , Antunes AF and Nunes VC , Insect sensory system, in Encyclopedia of Animal Cognition and Behavior, ed. by Vonk J and Shackelford TK . Springer International Publishing, New York, USA, pp. 3520–3532 (2022).

[7] Yan H , Insect olfactory neurons: receptors, development and function. Curr Opin Insect Sci 67:101288 (2024).39490981 10.1016/j.cois.2024.101288

[8] Lehane MJ , The biology of blood‐sucking in insects. Cambridge University Press, New York, USA (2005).

[9] Ignell R , Lazzari C , Lorenzo M and Hill S , Sensory ecology of disease vectors. Wageningen Academic Publishers, Wageningen, the Netherlands (2022). 37285450

[10] Shannon DM , Richardson N , Lahondère C and Peach D , Mosquito floral visitation and pollination. Curr Opin Insect Sci 64:101230 (2024). 10.1016/j.cois.2024.101230 38971524

[11] Hinze A , Hill S and Ignell R , Odour‐mediated host selection and discrimination in mosquitoes, in Sensory Ecology of Disease Vectors, ed. by Ignell R , Lazzari CR , Lorenzo MG and Hill SR . Wageningen Academic Publishers, Wageningen, the Netherlands, pp. 253–276 (2022).

[12] Vinauger C and Chandrasegaran K , Context‐specific variation in life history traits and behavior of *Aedes aegypti* mosquitoes. Front Insect Sci 4:1426715 (2024). 39386346 10.3389/finsc.2024.1426715 PMC11461241

[13] Booth W , Schal C and Vargo EL , Population genetics of bed bugs, in Advances in the Biology and Management of the Modern Bed Bug, ed. by Doggett SL , Miller DM and Lee CY . John Wiley & Sons Ltd, Oxford, UK, pp. 173–182 (2018).

[14] Usinger RL , Monograph of Cimicidae (Hemiptera, Heteroptera). Entomological Society of America, Lanham‐MD (1966).

[15] Evison SEF , Hentley WT , Wilson R and Siva‐Jothy MT , Bed bug biology, in Advances in the Biology and Management of the Modern Bed Bug, ed. by Doggett SL , Miller DM and Lee CY . John Wiley & Sons Ltd, Oxford, UK, pp. 149–161 (2018).

[16] Balvín O , Chajma P and Naylor R , Age structure of bed bug (Heteroptera: Cimicidae) aggregations affects the nymphal feeding success. Parasit Vectors 12:400 (2019). 31409390 10.1186/s13071-019-3659-5 PMC6693277

[17] Crawley SE , Borden JH , Ritchey JP and Haynes KF , First instar and adult male bed bugs, *Cimex lectularius* (Hemiptera: Cimicidae), increase feeding activity in the presence of adult females. Parasit Vectors 17:293 (2024). 38978105 10.1186/s13071-024-06289-3 PMC11232164

[18] Saenz VL , Santangelo RG , Vargo EL and Schal C , Group living accelerates bed bug (Hemiptera: Cimicidae) development. J Med Entomol 51:293–295 (2014). 24605482 10.1603/me13080

[19] Benoit JB , Del Grosso NA , Yoder JA and Denlinger DL , Resistance to dehydration between bouts of blood feeding in the bed bug, *Cimex lectularius*, is enhanced by water conservation, aggregation, and quiescence. Am J Trop Med Hyg 76:987–993 (2007). 17488928

[20] Marx R , Host‐finding and the importance of specific scents in the biology of *Cimex lectularius* . Z Parasitenkd 17:41–72 (1955).13248063

[21] Rivnay E , Studies in tropisms of the bed bug *Cimex lectularius* L. Parasitology 24:121–136 (1932).

[22] Aboul‐Nasr AE and Erakey MAS , Behaviour and sensory physiology of the bed‐bug, *Cimex lectularius* L., to some environmental factors: chemoreception (Hemiptera: Cimicidae). A R E 52:353–362 (1968).

[23] Gries R , Britton R , Holmes M , Zhai H , Draper J and Gries G , Bed bug aggregation pheromone finally identified. Angew Chem Int Ed 54:1135–1138 (2015). 10.1002/anie.201409890 25529634

[24] Olson JF , Vers LMV , Moon RD and Kells SA , Two compounds in bed bug feces are sufficient to elicit off‐host aggregation by bed bugs, *Cimex lectularius* . Pest Manag Sci 73:198–205 (2017). 27060680 10.1002/ps.4286

[25] Ulrich KR , Kramer M and Feldlaufer MF , Ability of bed bug (Hemiptera: Cimicidae) defensive secretions (*E*)‐2‐hexenal and (*E*)‐2‐octenal to attract adults of the common bed bug *Cimex lectularius* . Physiol Entomol 41:103–110 (2016).

[26] Choe DH , Park H , Vo C and Knyshov A , Chemically mediated arrestment of the bed bug, *Cimex lectularius*, by volatiles associated with exuviae of conspecifics. PLoS One 11:e0159520 (2016). 27434044 10.1371/journal.pone.0159520 PMC4951025

[27] Knudsen JT and Ignell R , Semiochemicals modulating bed bug behaviour. Curr Opin Insect Sci 64:101207 (2024). 38821142 10.1016/j.cois.2024.101207

[28] Akhoundi M , Chebbah D , Elissa N , Brun S , Jan J , Lacaze I *et al*., Volatile organic compounds: a promising tool for bed bug detection. Int J Environ Res Public Health 20:5214 (2023). 36982123 10.3390/ijerph20065214 PMC10048870

[29] Weeks E , Logan J , Birkett M , Caulfield J , Gezan S , Welham S *et al*., Electrophysiologically and behaviourally active semiochemicals identified from bed bug refuge substrate. Sci Rep 10:4590 (2020). 32165700 10.1038/s41598-020-61368-6 PMC7067832

[30] Harraca V , Ignell R , Löfstedt C and Ryne C , Characterization of the antennal olfactory system of the bed bug (*Cimex lectularius*). Chem Senses 35:195–204 (2010). 20032111 10.1093/chemse/bjp096

[31] Harraca V , Ryne C , Birgersson G and Ignell R , Smelling your way to food: can bed bugs use our odour? J Exp Biol 215:623–629 (2012). 22279069 10.1242/jeb.065748

[32] Liu F and Liu N , Human odorant reception in the common bed bug, *Cimex lectularius* . Sci Rep 5:15558 (2015). 26522967 10.1038/srep15558 PMC4629130

[33] Liu F , Chen Z and Liu N , Molecular basis of olfactory chemoreception in the common bed bug, *Cimex lectularius* . Sci Rep 7:45531 (2017). 28383033 10.1038/srep45531 PMC5382537

[34] Liu F , Xiong C and Liu N , Chemoreception to aggregation pheromones in the common bed bug, *Cimex lectularius* . Insect Biochem Mol Biol 82:62–73 (2017). 28167332 10.1016/j.ibmb.2017.01.012

[35] Liu F , Chen Z , Ye Z and Liu N , The olfactory chemosensation of hematophagous hemipteran insects. Front Physiol 12:703768 (2021). 34434117 10.3389/fphys.2021.703768 PMC8382127

[36] Hayes CC and Schal C , Review on the impacts of indoor vector control on domiciliary pests: good intentions challenged by harsh realities. Proc R Soc B 291:20240609 (2024). 10.1098/rspb.2024.0609 PMC11265923 39043243

[37] Sierras A and Schal C , Comparison of ingestion and topical application of insecticides against the common bed bug, *Cimex lectularius* (Hemiptera: Cimicidae). Pest Manag Sci 73:521–527 (2017). 27766740 10.1002/ps.4464 PMC5288133

[38] DeVries ZC , Saveer AM , Mick R and Schal C , Bed bug (Hemiptera: Cimicidae) attraction to human odors: validation of a two‐choice olfactometer. J Med Entomol 56:362–367 (2019). 30423171 10.1093/jme/tjy202 PMC7182910

[39] Anderson JF , Ferrandino FJ , McKnight S , Nolen J and Miller J , A carbon dioxide, heat and chemical lure trap for the bedbug, *Cimex lectularius* . Med Vet Entomol 23:99–105 (2009). 19499616 10.1111/j.1365-2915.2008.00790.x

[40] Wang C , Gibb T , Bennett GW and McKnight S , Bed bug (Heteroptera: Cimicidae) attraction to pitfall traps baited with carbon dioxide, heat, and chemical lure. J Econ Entomol 102:1580–1585 (2009). 19736771 10.1603/029.102.0423

[41] Miles LS , Verrelli BC , Adams R , Francioli YZ , Card DC , Balvin O *et al*., Were bed bugs the first urban pest insect? Genome‐wide patterns of bed bug demography mirror global human expansion. Biol Lett 5:20250061 (2025). 10.1098/rsbl.2025.0061 PMC12115845 40425045

[42] Scharf I , The multifaceted effects of starvation on arthropod behaviour. Anim Behav 119:37–48 (2016).

[43] Gadenne C , Barrozo RB and Anton S , Plasticity in insect olfaction: to smell or not to smell? Annu Rev Entomol 61:317–333 (2016). 26982441 10.1146/annurev-ento-010715-023523

[44] Force E , Suray C , Monsempes C , Fuentes A , Maria A and Debernard S , Modulation of the sex pheromone detection by nutritional and hormonal signals in a male insect. J Exp Biol 228:JEB249807 (2025). 39817435 10.1242/jeb.249807

[45] Davey KG , Symposium on reproduction of arthropods of medical and veterinary importance. VI. Reproduction in the females of some hematophagous insects. J Med Entomol 11:40–45 (1974). 4597471 10.1093/jmedent/11.1.40

[46] Barrozo RB , Reisenman CE , Guerenstein P , Lazzari CR and Lorenzo MG , An inside look at the sensory biology of triatomines. J Insect Physiol 97:3–19 (2017). 27840287 10.1016/j.jinsphys.2016.11.003

[47] Kong D , Wang Z , Guo H , Lin T , Jiang D , Qiu H *et al*., Identification of carbon dioxide receptors in the tropical bed bug, *Cimex hemipterus* (Hemiptera: Cimicidae). Insect Sci 0:1–15 (2025). 10.1111/1744-7917.70120 40619933

[48] Dormont L , Bessière LM and Cohuet A , Human skin volatiles: a review. J Chem Ecol 39:569–578 (2013). 23615881 10.1007/s10886-013-0286-z

[49] Penn DJ , Oberzaucher E , Grammer K , Fischer G , Soini HA , Wiesler D *et al*., Individual and gender fingerprints in human body odour. J R Soc Interface 4:331–340 (2007). 17251141 10.1098/rsif.2006.0182 PMC2359862

[50] Chen H , Zhao Q , Zhong Q , Duan C , Krutmann J , Wang J *et al*., Skin microbiome, metabolome and skin phenome, from the perspectives of skin as an ecosystem. Phenomics 2:363–382 (2022). 36939800 10.1007/s43657-022-00073-y PMC9712873

[51] Benoit JB , Adelman ZN , Reinhardt K , Dolan A , Poelchau M , Jennings EC *et al*., Unique features of a global human ectoparasite identified through sequencing of the bed bug genome. Nat Commun 7:10165 (2016). 26836814 10.1038/ncomms10165 PMC4740739

[52] McMeniman CJ , Corfas RA , Matthews BJ , Ritchie SA and Vosshall LB , Multimodal integration of carbon dioxide and other sensory cues drives mosquito attraction to humans. Cell 156:1060–1071 (2014). 24581501 10.1016/j.cell.2013.12.044 PMC4007582

[53] Galizia CG , Olfactory coding in the insect brain: data and conjectures. Eur J Neurosci 39:1784–1795 (2014). 24698302 10.1111/ejn.12558 PMC4237541

[54] Osborne LC , Palmer SE , Lisberger SG and Bialek W , The neural basis for combinatorial coding in a cortical population response. J Neurosci 28:13522–13531 (2008). 19074026 10.1523/JNEUROSCI.4390-08.2008 PMC2693376

[55] Fésűs L , Jobbágy A , Kiss N , Horváth E , Avci P , Lukács A *et al*., Dermatologic aspects of bed bug epidemic: an atlas of differential diagnosis. Postepy Dermatol Alergol 38:184–192 (2021). 36751539 10.5114/ada.2021.106194 PMC9880782

[56] Cooper R , Bed bugs – still more questions than answers: a need for research and public awareness. Am Entomol 5:111–112 (2006).

[57] Gaire S , Principato S , Schal C and DeVries ZC , Histamine excretion by the common bed bug (Hemiptera: Cimicidae). J Med Entomol 59:1898–1904 (2022). 36086827 10.1093/jme/tjac131 PMC9667729

[58] Principato S , Romero A , Lee CY , Campbell K , Choe DH , Schal C *et al*., Histamine excretion in common indoor and hematophagous arthropods. J Med Entomol 60:1269–1277 (2023). 37619246 10.1093/jme/tjad103 PMC10645371

[59] Weeks EN , Birkett MA , Cameron MM , Pickett JA and Logan JG , Semiochemicals of the common bed bug, *Cimex lectularius* L. (Hemiptera: Cimicidae), and their potential for use in monitoring and control. Pest Manag Sci 67:10–20 (2011). 20859928 10.1002/ps.2024

[60] Wang C , Tsai WT , Cooper R and White J , Effectiveness of bed bug monitors for detecting and trapping bed bugs in apartments. J Econ Entomol 104:274–278 (2011). 21404868 10.1603/ec10141

[61] Vail K and Chandler J , Bed bug (Hemiptera: Cimicidae) detection in low‐income, high‐rise apartments using four or fewer passive monitors. J Econ Entomol 110:1187–1194 (2017). 28369372 10.1093/jee/tox053

[62] Hottel BA , Pereira RM , Gezan SA and Koehler PG , Sticky trap design considerations for entrapping bed bugs. Insects 10:177 (2019). 31248145 10.3390/insects10060177 PMC6628081

[63] Potter MF , The history of bed bug management. Am Entomol 57:14–25 (2011).

[64] Saveer AM , DeVries ZC , Santangelo RG and Schal C , Mating and starvation modulate feeding and host‐seeking responses in female bed bugs, *Cimex lectularius* . Sci Rep 11:1915 (2021). 33479298 10.1038/s41598-021-81271-y PMC7820594

