# Supplementary material for: Bed bug preferences for host odor or aggregation odor are differentially modulated by physiological state in various odorscapes
Source: Pest Manag Sci. 2025 Oct 15;82(2):1426–36. doi: 10.1002/ps.70291 (PMC12790647; doi:10.1002/ps.70291)
Supplement: Supplementary file 1 — Data S1. Supporting Information. [file PS-82-1426-s001.docx]

Supplementary Material for “Bed bug preferences for Host skin odor or aggregation odor are differentially modulated by physiological state in various odorscapes”

Ayako Wada-Katsumata,^a*^ Christopher C. Hayes,^a^ Charles Kwadha,^a^ Alexander Ko^b^ and Coby Schal^a*^

**Correspondence to: A Wada-Katsumata or C Schal, Department of Entomology and Plant Pathology, North Carolina State University, Raleigh, NC, USA, E-mail:* [*akatsum@ncsu.edu*](mailto:akatsum@ncsu.edu) *or* [*coby@ncsu.edu*](mailto:coby@ncsu.edu)

a *Department of Entomology and Plant Pathology, North Carolina State University, Raleigh, NC, USA*

b *Envu, 5000 Centregreen Way, Suite 400, Cary, NC, USA*

This SM contains statistical analyses results:

Table S1. Full Factorial Repeated Measures ANOVA and Tukey’s HSD for Experiment 1 (Figure 2)

Table S2. Full Factorial Repeated Measures ANOVA and Tukey’s HSD for Experiment 2 (Figure 3)

Table S3. Full Factorial Repeated Measures ANOVA and Tukey’s HSD for Experiment 3 (Figure 4)

Table S4. Full Factorial Repeated Measures ANOVA and Tukey’s HSD for Experiments 4-6 (Figure 5)

Table S5. One-way ANOVA and Tukey’s HSD for Figure 6

| Table S1. Full Factorial Repeated Measures ANOVA and Tukey’s HSD for Experiment 1 (Figure 2) | | | | | | |
| --- | --- | --- | --- | --- | --- | --- |
|  | | | | | | |
| A. Fed  Aggregation odor vs Aggregation odor | | | | | | |
|  | ANOVA table | Number of parameters | DF | DFDen | F Ratio | P value (Prob>F) |
|  | Pitfall shelter | 1 | 1 | 4 | F (1, 4) = 0.5435 | 0.5019 |
|  | Time | 4 | 4 | 16 | F (2.018, 8.073) = 25.60 | < 0.0001 |
|  | Pitfall shelter x Time | 4 | 4 | 16 | F (2.018, 8.073) = 2.262 | 0.1078 |
| B. Unfed  Aggregation odor vs Aggregation odor | | | | | | |
|  | ANOVA table | Number of parameters | DF | DFDen | F Ratio | P value (Prob>F) |
|  | Pitfall shelter | 1 | 1 | 4 | F (1, 4) = 0.2579 | 0.6383 |
|  | Time | 4 | 4 | 16 | F (1.314, 5.255) = 58.78 | < 0.0001 |
|  | Pitfall shelter x Time | 4 | 4 | 16 | F (1.314, 5.255) = 0.7167 | 0.5928 |
| C. Fed  Host skin odor vs Host skin odor | | | | | | |
|  | ANOVA table | Number of parameters | DF | DFDen | F Ratio | P value (Prob>F) |
|  | Pitfall shelter | 1 | 1 | 4 | F (1, 4) = 0.2069 | 0.6728 |
|  | Time | 4 | 4 | 16 | F (1.500, 5.999) = 56.31 | < 0.0001 |
|  | Pitfall shelter x Time | 4 | 4 | 16 | F (1.500, 5.999) = 1.692 | 0.2009 |
| D. Unfed  Host skin odor vs Host skin odor | | | | | | |
|  | ANOVA table | Number of parameters | DF | DFDen | F Ratio | P value (Prob>F) |
|  | Pitfall shelter | 1 | 1 | 4 | F (1, 4) = 0.1250 | 0.7415 |
|  | Time | 4 | 4 | 16 | F (1.419, 5.677) = 23.60 | < 0.0001 |
|  | Pitfall shelter x Time | 4 | 4 | 16 | F (1.419, 5.677) = 1.034 | 0.4198 |

| Table S2. Full Factorial Repeated Measures ANOVA and Tukey’s HSD for Experiment 2 (Figure 3) | | | | | | |
| --- | --- | --- | --- | --- | --- | --- |
|  | | | | | | |
| A. Fed  Aggregation odor vs No odor | | | | | | |
|  | ANOVA table | Number of parameters | DF | DFDen | F Ratio | P value (Prob>F) |
|  | Pitfall shelter | 1 | 1 | 4 | F (1, 4) = 431.0 | < 0.0001 |
|  | Time | 4 | 4 | 16 | F (2.110, 8.442) = 109.7 | < 0.0001 |
|  | Pitfall shelter x Time | 4 | 4 | 16 | F (2.110, 8.442) = 63.38 | < 0.0001 |
|  |  |  |  |  | Time | P value (Prob>\|t\|) |
|  |  |  |  |  | 1hr | 0.0003 |
|  |  |  |  |  | 3hr | < 0.0001 |
|  |  |  |  |  | 6hr | < 0.0001 |
|  |  |  |  |  | 12hr | < 0.0001 |
|  |  |  |  |  | 24h | < 0.0001 |
| B. Unfed  Aggregation odor vs No odor | | | | | | |
|  | ANOVA table | Number of parameters | DF | DFDen | F Ratio | P value (Prob>F) |
|  | Pitfall shelter | 1 | 1 | 4 | F (1, 4) = 95.07 | 0.0006 |
|  | Time | 4 | 4 | 16 | F (1.421, 5.682) = 76.76 | < 0.0001 |
|  | Pitfall shelter x Time | 4 | 4 | 16 | F (1.421, 5.682) = 33.15 | < 0.0001 |
|  |  |  |  |  | Time | P value (Prob>\|t\|) |
|  |  |  |  |  | 1hr | 0.0876 |
|  |  |  |  |  | 3hr | 0.0020 |
|  |  |  |  |  | 6hr | < 0.0001 |
|  |  |  |  |  | 12hr | < 0.0001 |
|  |  |  |  |  | 24h | < 0.0001 |
| C. Fed  Host skin odor vs No odor | | | | | | |
|  | ANOVA table | Number of parameters | DF | DFDen | F Ratio | P value (Prob>F) |
|  | Pitfall shelter | 1 | 1 | 4 | F (1, 4) = 48.52 | 0.0022 |
|  | Time | 4 | 4 | 16 | F (2.463, 9.854) = 29.39 | < 0.0001 |
|  | Pitfall shelter x Time | 4 | 4 | 16 | F (2.463, 9.854) = 9.371 | 0.0004 |
|  |  |  |  |  | Time | P value (Prob>\|t\|) |
|  |  |  |  |  | 1hr | 0.8462 |
|  |  |  |  |  | 3hr | 0.0044 |
|  |  |  |  |  | 6hr | 0.0005 |
|  |  |  |  |  | 12hr | 0.0001 |
|  |  |  |  |  | 24h | 0.0001 |
| D. Unfed  Host skin odor vs No odor | | | | | | |
|  | ANOVA table | Number of parameters | DF | DFDen | F Ratio | P value (Prob>F) |
|  | Pitfall shelter | 1 | 1 | 4 | F (1, 4) = 274.8 | < 0.0001 |
|  | Time | 4 | 4 | 16 | F (1.594, 6.378) = 22.61 | < 0.0001 |
|  | Pitfall shelter x Time | 4 | 4 | 16 | F (1.594, 6.378) = 11.50 | 0.0001 |
|  |  |  |  |  | Time | P value (Prob>\|t\|) |
|  |  |  |  |  | 1hr | 0.0008 |
|  |  |  |  |  | 3hr | < 0.0001 |
|  |  |  |  |  | 6hr | < 0.0001 |
|  |  |  |  |  | 12hr | < 0.0001 |
|  |  |  |  |  | 24h | < 0.0001 |

| Table S3. Full Factorial Repeated Measures ANOVA and Tukey’s HSD for Experiment 3 (Figure 4) | | | | | | |
| --- | --- | --- | --- | --- | --- | --- |
|  | | | | | | |
| 2 days  Aggregation odor vs Host skin odor | | | | | | |
|  | ANOVA table | Number of parameters | DF | DFDen | F Ratio | P value (Prob>F) |
|  | Pitfall shelter | 1 | 1 | 4 | F (1, 4) = 56.51 | 0.0017 |
|  | Time | 4 | 4 | 16 | F (1.318, 5.271) = 11.64 | 0.0001 |
|  | Pitfall shelter x Time | 4 | 4 | 16 | F (1.318, 5.271) = 11.46 | 0.0001 |
|  |  |  |  |  | Time | P value (Prob>\|t\|) |
|  |  |  |  |  | 1hr | 0.0056 |
|  |  |  |  |  | 3hr | < 0.0001 |
|  |  |  |  |  | 6hr | < 0.0001 |
|  |  |  |  |  | 12hr | < 0.0001 |
|  |  |  |  |  | 24h | < 0.0001 |
| 4 days  Aggregation odor vs Host skin odor | | | | | | |
|  | ANOVA table | Number of parameters | DF | DFDen | F Ratio | P value (Prob>F) |
|  | Pitfall shelter | 1 | 1 | 4 | F (1, 4) = 96.36 | 0.0006 |
|  | Time | 4 | 4 | 16 | F (1.252, 5.008) = 18.79 | < 0.0001 |
|  | Pitfall shelter x Time | 4 | 4 | 16 | F (1.252, 5.008) = 5.636 | 0.0050 |
|  |  |  |  |  | Time | P value (Prob>\|t\|) |
|  |  |  |  |  | 1hr | 0.0030 |
|  |  |  |  |  | 3hr | < 0.0001 |
|  |  |  |  |  | 6hr | < 0.0001 |
|  |  |  |  |  | 12hr | < 0.0001 |
|  |  |  |  |  | 24h | < 0.0001 |
| 6 days  Aggregation odor vs Host skin odor | | | | | | |
|  | ANOVA table | Number of parameters | DF | DFDen | F Ratio | P value (Prob>F) |
|  | Pitfall shelter | 1 | 1 | 4 | F (1, 4) = 5.362 | 0.0815 |
|  | Time | 4 | 4 | 16 | F (1.594, 6.378) = 12.88 | < 0.0001 |
|  | Pitfall shelter x Time | 4 | 4 | 16 | F (1.594, 6.378) = 0.07303 | 0.9894 |
|  |  |  |  |  | Time | P value (Prob>\|t\|) |
|  |  |  |  |  | 1hr | 0.6635 |
|  |  |  |  |  | 3hr | 0.7709 |
|  |  |  |  |  | 6hr | 0.8622 |
|  |  |  |  |  | 12hr | 0.8622 |
|  |  |  |  |  | 24h | 0.9296 |
| 8 days  Aggregation odor vs Host skin odor | | | | | | |
|  | ANOVA table | Number of parameters | DF | DFDen | F Ratio | P value (Prob>F) |
|  | Pitfall shelter | 1 | 1 | 4 | F (1, 4) = 8.862 | 0.0409 |
|  | Time | 4 | 4 | 16 | F (1.378, 5.513) = 18.02 | < 0.0001 |
|  | Pitfall shelter x Time | 4 | 4 | 16 | F (1.378, 5.513) = 0.5811 | 0.6807 |
|  |  |  |  |  | Time | P value (Prob>\|t\|) |
|  |  |  |  |  | 1hr | 0.9999 |
|  |  |  |  |  | 3hr | 0.9969 |
|  |  |  |  |  | 6hr | 0.8057 |
|  |  |  |  |  | 12hr | 0.9775 |
|  |  |  |  |  | 24h | 0.3469 |
| 10 days  Aggregation odor vs Host skin odor | | | | | | |
|  | ANOVA table | Number of parameters | DF | DFDen | F Ratio | P value (Prob>F) |
|  | Pitfall shelter | 1 | 1 | 4 | F (1, 4) = 4.414 | 0.1036 |
|  | Time | 4 | 4 | 16 | F (2.394, 9.577) = 18.05 | < 0.0001 |
|  | Pitfall shelter x Time | 4 | 4 | 16 | F (2.394, 9.577) = 3.348 | 0.0358 |
|  |  |  |  |  | Time | P value (Prob>\|t\|) |
|  |  |  |  |  | 1hr | 1.0000 |
|  |  |  |  |  | 3hr | 0.9997 |
|  |  |  |  |  | 6hr | 0.9801 |
|  |  |  |  |  | 12hr | 0.4296 |
|  |  |  |  |  | 24h | 0.0593 |
| 12 days  Aggregation odor vs Host skin odor | | | | | | |
|  | ANOVA table | Number of parameters | DF | DFDen | F Ratio | P value (Prob>F) |
|  | Pitfall shelter | 1 | 1 | 4 | F (1, 4) = 42.92 | 0.0028 |
|  | Time | 4 | 4 | 16 | F (1.687, 6.747) = 10.73 | 0.0002 |
|  | Pitfall shelter x Time | 4 | 4 | 16 | F (1.687, 6.747) = 2.204 | 0.1147 |
|  |  |  |  |  | Time | P value (Prob>\|t\|) |
|  |  |  |  |  | 1hr | 0.2901 |
|  |  |  |  |  | 3hr | 0.0176 |
|  |  |  |  |  | 6hr | 0.0058 |
|  |  |  |  |  | 12hr | 0.0019 |
|  |  |  |  |  | 24h | 0.0011 |
| 14 days  Aggregation odor vs Host skin odor | | | | | | |
|  | ANOVA table | Number of parameters | DF | DFDen | F Ratio | P value (Prob>F) |
|  | Pitfall shelter | 1 | 1 | 4 | F (1, 4) = 217.8 | 0.0001 |
|  | Time | 4 | 4 | 16 | F (1.781, 7.125) = 13.04 | < 0.0001 |
|  | Pitfall shelter x Time | 4 | 4 | 16 | F (1.781, 7.125) = 17.03 | < 0.0001 |
|  |  |  |  |  | Time | P value (Prob>\|t\|) |
|  |  |  |  |  | 1hr | 0.9994 |
|  |  |  |  |  | 3hr | 0.0001 |
|  |  |  |  |  | 6hr | < 0.0001 |
|  |  |  |  |  | 12hr | < 0.0001 |
|  |  |  |  |  | 24h | < 0.0001 |

| Table S4. Full Factorial Repeated Measures ANOVA and Tukey’s HSD for Experiment 4, 5 and 6 (Figure 5A, B and C) | | | | | | |
| --- | --- | --- | --- | --- | --- | --- |
|  | | | | | | |
| A. Fed  Host odor vs Aggregation odor + Host odor | | | | | | |
|  | ANOVA table | Number of parameters | DF | DFDen | F Ratio | P value (Prob>F) |
|  | Pitfall shelter | 1 | 1 | 4 | F (1, 4) = 82.19 | 0.0008 |
|  | Time | 4 | 4 | 16 | F (1.686, 6.745) = 24.56 | < 0.0001 |
|  | Pitfall shelter x Time | 4 | 4 | 16 | F (1.686, 6.745) = 15.04 | < 0.0001 |
|  |  |  |  |  | Time | P value (Prob>\|t\|) |
|  |  |  |  |  | 1hr | 0.4502 |
|  |  |  |  |  | 3hr | 0.0124 |
|  |  |  |  |  | 6hr | 0.0002 |
|  |  |  |  |  | 12hr | < 0.0001 |
|  |  |  |  |  | 24h | < 0.0001 |
| A. Unfed  Host odor vs Aggregation odor + Host odor | | | | | | |
|  | ANOVA table | Number of parameters | DF | DFDen | F Ratio | P value (Prob>F) |
|  | Pitfall shelter | 1 | 1 | 4 | F (1, 4) = 0.002817 | 0.9602 |
|  | Time | 4 | 4 | 16 | F (1.441, 5.765) = 13.56 | < 0.0001 |
|  | Pitfall shelter x Time | 4 | 4 | 16 | F (1.441, 5.765) = 0.5446 | 0.7055 |
|  |  |  |  |  | Time | P value (Prob>\|t\|) |
|  |  |  |  |  | 1hr | 0.9991 |
|  |  |  |  |  | 3hr | 1.0000 |
|  |  |  |  |  | 6hr | 1.0000 |
|  |  |  |  |  | 12hr | 1.0000 |
|  |  |  |  |  | 24h | 1.0000 |

| B. Fed  Aggregation odor vs Aggregation odor + Host odor | | | | | | |
| --- | --- | --- | --- | --- | --- | --- |
|  | ANOVA table | Number of parameters | DF | DFDen | F Ratio | P value (Prob>F) |
|  | Pitfall shelter | 1 | 1 | 4 | F (1, 4) = 10.61 | 0.0312 |
|  | Time | 4 | 4 | 16 | F (1.999, 7.995) = 14.57 | 0.0079 |
|  | Pitfall shelter x Time | 4 | 4 | 16 | F (1.999, 7.995) = 5.056 | < 0.0001 |
|  |  |  |  |  | Time | P value (Prob>\|t\|) |
|  |  |  |  |  | 1hr | 0.4633 |
|  |  |  |  |  | 3hr | 0.2353 |
|  |  |  |  |  | 6hr | 0.0618 |
|  |  |  |  |  | 12hr | 0.0516 |
|  |  |  |  |  | 24h | 0.0516 |
| B. Unfed  Aggregation odor vs Aggregation odor + Host odor | | | | | | |
|  | ANOVA table | Number of parameters | DF | DFDen | F Ratio | P value (Prob>F) |
|  | Pitfall shelter | 1 | 1 | 4 | F (1, 4) = 19.20 | 0.0119 |
|  | Time | 4 | 4 | 16 | F (1.527, 6.107) = 4.590 | 0.0117 |
|  | Pitfall shelter x Time | 4 | 4 | 16 | F (1.527, 6.107) = 2.857 | 0.0581 |
|  |  |  |  |  | Time | P value (Prob>\|t\|) |
|  |  |  |  |  | 1hr | 1.0000 |
|  |  |  |  |  | 3hr | 0.8577 |
|  |  |  |  |  | 6hr | 0.1815 |
|  |  |  |  |  | 12hr | 0.0750 |
|  |  |  |  |  | 24h | 0.0290 |

| C. Fed  Aggregation odor vs Aggregation odor + Host odor + CO_2_ | | | | | | |
| --- | --- | --- | --- | --- | --- | --- |
|  | ANOVA table | Number of parameters | DF | DFDen | F Ratio | P value (Prob>F) |
|  | Pitfall shelter | 1 | 1 | 4 | F (1, 4) = 1191 | < 0.0001 |
|  | Time | 2 | 2 | 8 | F (1.048, 4.190) = 1.696 | 0.2433 |
|  | Pitfall shelter x Time | 2 | 2 | 8 | F (1.048, 4.190) = 12.04 | 0.0039 |
|  |  |  |  |  | Time | P value (Prob>\|t\|) |
|  |  |  |  |  | 1hr | < 0.0001 |
|  |  |  |  |  | 3hr | < 0.0001 |
|  |  |  |  |  | 6hr | < 0.0001 |
| C. Unfed  Aggregation odor vs Aggregation odor + Host odor + CO_2_ | | | | | | |
|  | ANOVA table | Number of parameters | DF | DFDen | F Ratio | P value (Prob>F) |
|  | Pitfall shelter | 1 | 1 | 4 | F (1, 4) = 600.2 | < 0.0001 |
|  | Time | 2 | 2 | 8 | F (1.226, 4.902) = 5.250 | 0.0007 |
|  | Pitfall shelter x Time | 2 | 2 | 8 | F (1.226, 4.902) = 20.58 | 0.0350 |
|  |  |  |  |  | Time | P value (Prob>\|t\|) |
|  |  |  |  |  | 1hr | 0.0001 |
|  |  |  |  |  | 3hr | < 0.0001 |
|  |  |  |  |  | 6hr | < 0.0001 |

| Table S5. One-way ANOVA and Tukey’s HSD for Fig. 6 | | | | | | |
| --- | --- | --- | --- | --- | --- | --- |
|  |  |  |  |  |  |  |
| A. 6 hr time point  Preference for aggregation odor | | | | | | |
|  | ANOVA table | SS | DF | MS | F (DFn, DFd) | P value |
|  | Treatment | 1717 | 7 | 245.3 | F (7, 16) = 46.36 | < 0.0001 |
|  | Residual | 84.67 | 16 | 5.292 |  |  |
|  | Total | 1802 | 23 |  |  |  |
|  |  | Mean Diff. | 95% CI | Below threshold | Summary | P-value |
|  | Exp2Fed vs. Exp2Unfed | 6 | -0.5027 to 12.50 | No | ns | 0.0819 |
|  | Exp2Fed vs. Exp3Fed | -4 | -10.50 to 2.503 | No | ns | 0.4377 |
|  | Exp2Fed vs. Exp3Unfed | 17.67 | 11.16 to 24.17 | Yes | **** | < 0.0001 |
|  | Exp2Fed vs. Exp5Fed | 1.667 | -4.836 to 8.169 | No | ns | 0.9831 |
|  | Exp2Fed vs. Exp5Unfed | 11.67 | 5.164 to 18.17 | Yes | *** | 0.0003 |
|  | Exp2Fed vs. Exp6Fed | -4.667 | -11.17 to 1.836 | No | ns | 0.2685 |
|  | Exp2Fed vs. Exp6Unfed | 17.67 | 11.16 to 24.17 | Yes | **** | < 0.0001 |
|  | Exp2Unfed vs. Exp3Fed | -10 | -16.50 to -3.497 | Yes | ** | 0.0014 |
|  | Exp2Unfed vs. Exp3Unfed | 11.67 | 5.164 to 18.17 | Yes | *** | 0.0003 |
|  | Exp2Unfed vs. Exp5Fed | -4.333 | -10.84 to 2.169 | No | ns | 0.3467 |
|  | Exp2Unfed vs. Exp5Unfed | 5.667 | -0.8361 to 12.17 | No | ns | 0.1123 |
|  | Exp2Unfed vs. Exp6Fed | -10.67 | -17.17 to -4.164 | Yes | *** | 0.0007 |
|  | Exp2Unfed vs. Exp6Unfed | 11.67 | 5.164 to 18.17 | Yes | *** | 0.0003 |
|  | Exp3Fed vs. Exp3Unfed | 21.67 | 15.16 to 28.17 | Yes | **** | < 0.0001 |
|  | Exp3Fed vs. Exp5Fed | 5.667 | -0.8361 to 12.17 | No | ns | 0.1123 |
|  | Exp3Fed vs. Exp5Unfed | 15.67 | 9.164 to 22.17 | Yes | **** | < 0.0001 |
|  | Exp3Fed vs. Exp6Fed | -0.6667 | -7.169 to 5.836 | No | ns | > 0.9999 |
|  | Exp3Fed vs. Exp6Unfed | 21.67 | 15.16 to 28.17 | Yes | **** | < 0.0001 |
|  | Exp3Unfed vs. Exp5Fed | -16 | -22.50 to -9.497 | Yes | **** | < 0.0001 |
|  | Exp3Unfed vs. Exp5Unfed | -6 | -12.50 to 0.5027 | No | ns | 0.0819 |
|  | Exp3Unfed vs. Exp6Fed | -22.33 | -28.84 to -15.83 | Yes | **** | < 0.0001 |
|  | Exp3Unfed vs. Exp6Unfed | 0 | -6.503 to 6.503 | No | ns | > 0.9999 |
|  | Exp5Fed vs. Exp5Unfed | 10 | 3.497 to 16.50 | Yes | ** | 0.0014 |
|  | Exp5Fed vs. Exp6Fed | -6.333 | -12.84 to 0.1694 | No | ns | 0.0591 |
|  | Exp5Fed vs. Exp6Unfed | 16 | 9.497 to 22.50 | Yes | **** | < 0.0001 |
|  | Exp5Unfed vs. Exp6Fed | -16.33 | -22.84 to -9.831 | Yes | **** | < 0.0001 |
|  | Exp5Unfed vs. Exp6Unfed | 6 | -0.5027 to 12.50 | No | ns | 0.0819 |
|  | Exp6Fed vs. Exp6Unfed | 22.33 | 15.83 to 28.84 | Yes | **** | < 0.0001 |
| B. 6 hr time point  Preference for Host skin odor | | | | | | |
|  | ANOVA table | SS | DF | MS | F (DFn, DFd) | P value |
|  | Treatment | 615.8 | 5 | 123.2 | F (5, 12) = 26.08 | < 0.0001 |
|  | Residual | 56.67 | 12 | 4.722 |  |  |
|  | Total | 672.4 | 17 |  |  |  |
|  |  | Mean Diff. | 95% CI | Below threshold | Summary | P-value |
|  | Exp2Fed vs. Exp2Unfed | -11.67 | -17.63 to -5.707 | Yes | *** | 0.0003 |
|  | Exp2Fed vs. Exp3Fed | 3 | -2.960 to 8.960 | No | ns | 0.5614 |
|  | Exp2Fed vs. Exp3Unfed | -10.33 | -16.29 to -4.374 | Yes | *** | 0.0009 |
|  | Exp2Fed vs. Exp4Fed | 2 | -3.960 to 7.960 | No | ns | 0.8611 |
|  | Exp2Fed vs. Exp4Unfed | -6.333 | -12.29 to -0.3736 | Yes | * | 0.0351 |
|  | Exp2Unfed vs. Exp3Fed | 14.67 | 8.707 to 20.63 | Yes | **** | <0.0001 |
|  | Exp2Unfed vs. Exp3Unfed | 1.333 | -4.626 to 7.293 | No | ns | 0.9708 |
|  | Exp2Unfed vs. Exp4Fed | 13.67 | 7.707 to 19.63 | Yes | **** | < 0.0001 |
|  | Exp2Unfed vs. Exp4Unfed | 5.333 | -0.6264 to 11.29 | No | ns | 0.0897 |
|  | Exp3Fed vs. Exp3Unfed | -13.33 | -19.29 to -7.374 | Yes | **** | < 0.0001 |
|  | Exp3Fed vs. Exp4Fed | -1 | -6.960 to 4.960 | No | ns | 0.9917 |
|  | Exp3Fed vs. Exp4Unfed | -9.333 | -15.29 to -3.374 | Yes | ** | 0.0021 |
|  | Exp3Unfed vs. Exp4Fed | 12.33 | 6.374 to 18.29 | Yes | *** | 0.0002 |
|  | Exp3Unfed vs. Exp4Unfed | 4 | -1.960 to 9.960 | No | ns | 0.2829 |
|  | Exp4Fed vs. Exp4Unfed | -8.333 | -14.29 to -2.374 | Yes | ** | 0.0053 |

| 12 hr time point  Preference for aggregation odor | | | | | | |
| --- | --- | --- | --- | --- | --- | --- |
|  | ANOVA table | SS | DF | MS | F (DFn, DFd) | P value |
|  | Treatment | 1029 | 5 | 205.7 | F (5, 12) = 40.25 | < 0.0001 |
|  | Residual | 61.33 | 12 | 5.111 |  |  |
|  | Total | 1090 | 17 |  |  |  |
|  |  | Mean Diff. | 95% CI | Below threshold | Summary | P-value |
|  | Exp2Fed vs. Exp2Unfed | 4.333 | -1.867 to 10.53 | No | ns | 0.2481 |
|  | Exp2Fed vs. Exp3Fed | -3 | -9.200 to 3.200 | No | ns | 0.5989 |
|  | Exp2Fed vs. Exp3Unfed | 19 | 12.80 to 25.20 | Yes | **** | < 0.0001 |
|  | Exp2Fed vs. Exp5Fed | 1.667 | -4.534 to 7.867 | No | ns | 0.9385 |
|  | Exp2Fed vs. Exp5Unfed | 12 | 5.800 to 18.20 | Yes | *** | 0.0003 |
|  | Exp2Unfed vs. Exp3Fed | -7.333 | -13.53 to -1.133 | Yes | * | 0.0178 |
|  | Exp2Unfed vs. Exp3Unfed | 14.67 | 8.466 to 20.87 | Yes | **** | < 0.0001 |
|  | Exp2Unfed vs. Exp5Fed | -2.667 | -8.867 to 3.534 | No | ns | 0.702 |
|  | Exp2Unfed vs. Exp5Unfed | 7.667 | 1.466 to 13.87 | Yes | * | 0.0131 |
|  | Exp3Fed vs. Exp3Unfed | 22 | 15.80 to 28.20 | Yes | **** | < 0.0001 |
|  | Exp3Fed vs. Exp5Fed | 4.667 | -1.534 to 10.87 | No | ns | 0.1903 |
|  | Exp3Fed vs. Exp5Unfed | 15 | 8.800 to 21.20 | Yes | **** | < 0.0001 |
|  | Exp3Unfed vs. Exp5Fed | -17.33 | -23.53 to -11.13 | Yes | **** | < 0.0001 |
|  | Exp3Unfed vs. Exp5Unfed | -7 | -13.20 to -0.7997 | Yes | * | 0.0241 |
|  | Exp5Fed vs. Exp5Unfed | 10.33 | 4.133 to 16.53 | Yes | ** | 0.0013 |
| 12 hr time point  Preference for Host skin odor | | | | | | |
|  | ANOVA table | SS | DF | MS | F (DFn, DFd) | P value |
|  | Treatment | 827.1 | 5 | 165.4 | F (5, 12) = 29.78 | < 0.0001 |
|  | Residual | 66.67 | 12 | 5.556 |  |  |
|  | Total | 893.8 | 17 |  |  |  |
|  |  | Mean Diff. | 95% CI | Below threshold | Summary | P-value |
|  | Exp2Fed vs. Exp2Unfed | -12.33 | -18.80 to -5.869 | Yes | *** | 0.0004 |
|  | Exp2Fed vs. Exp3Fed | 4 | -2.464 to 10.46 | No | ns | 0.3581 |
|  | Exp2Fed vs. Exp3Unfed | -13 | -19.46 to -6.536 | Yes | *** | 0.0002 |
|  | Exp2Fed vs. Exp4Fed | 2.333 | -4.131 to 8.798 | No | ns | 0.823 |
|  | Exp2Fed vs. Exp4Unfed | -6.333 | -12.80 to 0.1309 | No | ns | 0.056 |
|  | Exp2Unfed vs. Exp3Fed | 16.33 | 9.869 to 22.80 | Yes | **** | < 0.0001 |
|  | Exp2Unfed vs. Exp3Unfed | -0.6667 | -7.131 to 5.798 | No | ns | 0.9992 |
|  | Exp2Unfed vs. Exp4Fed | 14.67 | 8.202 to 21.13 | Yes | **** | < 0.0001 |
|  | Exp2Unfed vs. Exp4Unfed | 6 | -0.4642 to 12.46 | No | ns | 0.0747 |
|  | Exp3Fed vs. Exp3Unfed | -17 | -23.46 to -10.54 | Yes | **** | < 0.0001 |
|  | Exp3Fed vs. Exp4Fed | -1.667 | -8.131 to 4.798 | No | ns | 0.9478 |
|  | Exp3Fed vs. Exp4Unfed | -10.33 | -16.80 to -3.869 | Yes | ** | 0.0018 |
|  | Exp3Unfed vs. Exp4Fed | 15.33 | 8.869 to 21.80 | Yes | **** | < 0.0001 |
|  | Exp3Unfed vs. Exp4Unfed | 6.667 | 0.2024 to 13.13 | Yes | * | 0.0419 |
|  | Exp4Fed vs. Exp4Unfed | -8.667 | -15.13 to -2.202 | Yes | ** | 0.0073 |

| 24 hr time point  Preference for aggregation odor | | | | | | |
| --- | --- | --- | --- | --- | --- | --- |
|  | ANOVA table | SS | DF | MS | F (DFn, DFd) | P value |
|  | Treatment | 660 | 5 | 132 | F (5, 12) = 21.41 | < 0.0001 |
|  | Residual | 74 | 12 | 6.167 |  |  |
|  | Total | 734 | 17 |  |  |  |
|  |  | Mean Diff. | 95% CI | Below threshold | Summary | P-value |
|  | Exp2Fed vs. Exp2Unfed | 4 | -2.811 to 10.81 | No | ns | 0.409 |
|  | Exp2Fed vs. Exp3Fed | -3 | -9.811 to 3.811 | No | ns | 0.6823 |
|  | Exp2Fed vs. Exp3Unfed | 13.67 | 6.856 to 20.48 | Yes | *** | 0.0002 |
|  | Exp2Fed vs. Exp5Fed | 1.667 | -5.144 to 8.477 | No | ns | 0.9577 |
|  | Exp2Fed vs. Exp5Unfed | 11.67 | 4.856 to 18.48 | Yes | *** | 0.001 |
|  | Exp2Unfed vs. Exp3Fed | -7 | -13.81 to -0.1895 | Yes | * | 0.0427 |
|  | Exp2Unfed vs. Exp3Unfed | 9.667 | 2.856 to 16.48 | Yes | ** | 0.0047 |
|  | Exp2Unfed vs. Exp5Fed | -2.333 | -9.144 to 4.477 | No | ns | 0.851 |
|  | Exp2Unfed vs. Exp5Unfed | 7.667 | 0.8562 to 14.48 | Yes | * | 0.0245 |
|  | Exp3Fed vs. Exp3Unfed | 16.67 | 9.856 to 23.48 | Yes | **** | < 0.0001 |
|  | Exp3Fed vs. Exp5Fed | 4.667 | -2.144 to 11.48 | No | ns | 0.2648 |
|  | Exp3Fed vs. Exp5Unfed | 14.67 | 7.856 to 21.48 | Yes | *** | 0.0001 |
|  | Exp3Unfed vs. Exp5Fed | -12 | -18.81 to -5.189 | Yes | *** | 0.0008 |
|  | Exp3Unfed vs. Exp5Unfed | -2 | -8.811 to 4.811 | No | ns | 0.9138 |
|  | Exp5Fed vs. Exp5Unfed | 10 | 3.189 to 16.81 | Yes | ** | 0.0036 |
|  | | | | | | |
|  | ANOVA table | SS | DF | MS | F (DFn, DFd) | P value |
|  | Treatment | 926 | 5 | 185.2 | F (5, 12) = 30.03 | < 0.0001 |
|  | Residual | 74 | 12 | 6.167 |  |  |
|  | Total | 1000 | 17 |  |  |  |
|  |  | Mean Diff. | 95% CI | Below threshold | Summary | P-value |
|  | Exp2Fed vs. Exp2Unfed | -13.33 | -20.14 to -6.523 | Yes | *** | 0.0003 |
|  | Exp2Fed vs. Exp3Fed | 5.667 | -1.144 to 12.48 | No | ns | 0.126 |
|  | Exp2Fed vs. Exp3Unfed | -11 | -17.81 to -4.189 | Yes | ** | 0.0016 |
|  | Exp2Fed vs. Exp4Fed | 4 | -2.811 to 10.81 | No | ns | 0.409 |
|  | Exp2Fed vs. Exp4Unfed | -5.333 | -12.14 to 1.477 | No | ns | 0.1629 |
|  | Exp2Unfed vs. Exp3Fed | 19 | 12.19 to 25.81 | Yes | **** | < 0.0001 |
|  | Exp2Unfed vs. Exp3Unfed | 2.333 | -4.477 to 9.144 | No | ns | 0.851 |
|  | Exp2Unfed vs. Exp4Fed | 17.33 | 10.52 to 24.14 | Yes | **** | < 0.0001 |
|  | Exp2Unfed vs. Exp4Unfed | 8 | 1.189 to 14.81 | Yes | * | 0.0186 |
|  | Exp3Fed vs. Exp3Unfed | -16.67 | -23.48 to -9.856 | Yes | **** | < 0.0001 |
|  | Exp3Fed vs. Exp4Fed | -1.667 | -8.477 to 5.144 | No | ns | 0.9577 |
|  | Exp3Fed vs. Exp4Unfed | -11 | -17.81 to -4.189 | Yes | ** | 0.0016 |
|  | Exp3Unfed vs. Exp4Fed | 15 | 8.189 to 21.81 | Yes | **** | < 0.0001 |
|  | Exp3Unfed vs. Exp4Unfed | 5.667 | -1.144 to 12.48 | No | ns | 0.126 |
|  | Exp4Fed vs. Exp4Unfed | -9.333 | -16.14 to -2.523 | Yes | ** | 0.0062 |
